# Supplementary material for: Inoculation with indigenous nitrogen-fixers enhances seedling growth and nutrient uptake in a greenhouse bioassay
Source: PLoS One. 2026 Apr 15;21(4):e0339012. doi: 10.1371/journal.pone.0339012 (PMC13082604; doi:10.1371/journal.pone.0339012)
Supplement: S1 Table — (DOCX) [file pone.0339012.s001.docx]

**Table S1. Indigenous Nitrogen Fixing Bacterial Isolates Used for the Preparation of Inoculum**

| **Plant Species** | **Isolation Source** | **Isolate ID Number** | **Molecular Identification (Basic Local Alignment Search Tool [BLAST])** | | |  |
| --- | --- | --- | --- | --- | --- | --- |
|  |  |  | **Isolate Identity** | **% Identity** | **NCBI Accession Number** | |
| *Rhanterium epapposum* | Soil | RhaS-1 | *Sphingomonas zeicaulis* strain 541 | 503/518(97%) | NR_152012 | |
|  |  | RhaS-2 | *Klebsiella pneumoniae* subsp. Rhinoscleromatis | 536/543(99%) | NR_114507 | |
|  |  | RhaS-4 | *Rhizobium pakistanense* strain BN-19 | 515/517(99%) | NR_145565 | |
|  |  | RhaS-6 | *Klebsiella pneumoniae* subsp. rhinoscleromatis | 537/543(99%) | NR_114507 | |
|  |  | RhaS-9 | *Rhizobium pakistanense* strain BN-19 | 514/517(99%) | NR_145565 | |
|  |  | RhaS-12 | *Rhizobium pakistanense* strain BN-19 | 515/517(99%) | NR_145565 | |
| *Farsetia aegyptia* | Soil | FarS-1 | *Rhizobium pakistanense* strain BN-19 | 501/502(99%) | NR_145565 | |
|  |  | FarS-4 | *Pseudomonas glareae* strain KMM 9500 | 529/539(98%) | NR_145562 | |
|  |  | FarS-8 | *Pseudomonas stutzeri* strain VKM B-975 | 536/537(99%) | NR_116489 | |
| *Haloxylon salicornicum* | Soil | HalS-1 | *Rhizobium subbaraonis* strain JC85 | 512/517(99%) | NR_108508 | |
|  |  | HalS-8 | *Pseudomonas stutzeri* strain VKM B-975 | 570/571(99%) | NR_116489 | |
|  |  | HalS- 9 | *Rhizobium subbaraonis* strain JC85 | 535/540(99%) | NR_108508 | |
|  |  | HalS-12 | *Pseudomonas songnenensis* strain NEAU-ST5-5 | 575/578(99%) | NR_148295 | |
|  |  | HalS-13 | *Rhizobium pakistanense* strain BN-19 | 535/540(99%) | NR_145565 | |
|  |  | HalS-14 | *Rhizobium subbaraonis* strain JC85 | 536/540(99%) | NR_108508 | |
|  |  | HalS-17 | *Massilia timonae* strain UR/MT95 | 562/565(99%) | NR_026014 | |
|  |  | HalS-19 | *Rhizobium pakistanense* strain BN-19 | 536/540(99%) | NR_145565 | |
|  |  | HalS-21 | Leifsonia shinshuensis strain DB 102 | 547/548(99%) | NR_043663 | |
|  |  | HalS-23 | *Rhizobium pakistanense* strain BN-19 | 536/540(99%) | NR_145565 | |
|  |  | HalS-24 | *Microbacterium assamensis* strain S2-48 | 548/548(100%) | NR_132711 | |
| *Vachellia pachyceras* | Root Nodule | Ac-1 | *Agrobacterium tumefaciens* strain IAM 12048 | 540/540(100%) | NR_041396 | |
|  |  | Ac-9 | *Agrobacterium tumefaciens* strain IAM 12048 | 540/540(100%) | NR_041396 | |
|  |  | Ac-10 | *Agrobacterium tumefaciens* strain IAM 12048 | 540/540(100%) | NR_041396 | |
|  |  | Ac-12 | *Agrobacterium tumefaciens* strain IAM 12048 | 540/540(100%) | NR_041396 | |
|  |  | Ac-22 | *Agrobacterium tumefaciens* strain IAM 12048 | 539/540(99%) | NR_041396 | |
|  |  | Ac-25 | *Agrobacterium tumefaciens* strain IAM 12048 | 538/540(99%) | NR_041396 | |
|  |  | Ac-30 | *Agrobacterium tumefaciens* strain IAM 12048 | 536/540(99%) | NR_041396 | |
|  |  | Ac-35 | *Agrobacterium tumefaciens* strain IAM 12048 | 539/540(99%) | NR_041396 | |
|  |  | Ac-39 | *Pseudomonas koreensis* strain Ps 9-14 | 530/532(99%) | NR_025228 | |
|  |  | Ac-40 | *Agrobacterium tumefaciens* strain IAM 12048 | 538/540(99%) | NR_041396 | |
|  |  | ACN-1 | *Pseudomonas koreensis* strain Ps 9-14 | 554/556(99%) | NR_025228 | |
|  |  | ACN-2 | *Enterobacter cloacae* strain ATCC 13047 | 574/577(99%) | NR_118568 | |
|  |  | ACN-3 | *Pseudomonas koreensis* strain Ps 9-14 | 531/533(99%) | NR_025228 | |
|  |  | ACN-9 | *Pseudomonas koreensis* strain Ps 9-14 | 536/538(99%) | NR_025228 | |
|  |  | ACN-10 | *Arthrobacter nitroguajacolicus* strain G2-1 | 515/515(100%) | NR_027199 | |
|  |  | ACN-12 | *Cellulomonas massiliensis* strain JC225 | 556/559(99%) | NR_125601 | |
|  |  | ACN-14 | *Cellulomonas massiliensis* strain JC225 | 556/559(99%) | NR_125601 | |
|  |  | ACN-17 | *Cellulomonas massiliensis* strain JC225 | 553/558(99%) | NR_125601 | |
|  |  | LTN-1 | *Bacillus simplex* strain LMG 11160 | 577/579(99%) | NR_114919 | |
