# Supplementary material for: Inoculation with indigenous nitrogen-fixers enhances seedling growth and nutrient uptake in a greenhouse bioassay
Source: PLoS One. 2026 Apr 15;21(4):e0339012. doi: 10.1371/journal.pone.0339012 (PMC13082604; doi:10.1371/journal.pone.0339012)
Supplement: S2 Table — (DOCX) [file pone.0339012.s002.docx]

**Table S2. Physio-Chemical Properties of Desert Soil Collected from KISR’s Station for Research and Innovation (KSRI)**

| **Physical Properties** | | | | | **Chemical Properties** | | | | | | | | | | | |
| --- | --- | --- | --- | --- | --- | --- | --- | --- | --- | --- | --- | --- | --- | --- | --- | --- |
| **Bulk Density** | **Particle Size Analysis (%)** | | | **Texture** | **pH** | **EC^*^ (mS/cm)** | **Ca** | **Mg** | **Na** | **K** | **P** | **Cl** | **Organic Matter** | **C** | **N** | **S** |
| **(g/cm^3^)** | **Sand** | **Clay** | **Silt** |  |  |  | **mg/kg** | | | | | | **%** | | | |
| 1.67 | 94 | 5 | 1 | Sand | 8.37 | 0.3 | 6,374 | 143 | 575 | 310 | 12 | 51 | 0.19 | 0.8 | 0.006 | 0.0004 |
