# Supplementary material for: Inoculation with indigenous nitrogen-fixers enhances seedling growth and nutrient uptake in a greenhouse bioassay
Source: PLoS One. 2026 Apr 15;21(4):e0339012. doi: 10.1371/journal.pone.0339012 (PMC13082604; doi:10.1371/journal.pone.0339012)
Supplement: Attachment — Submitted filename: PONE-D-25-64029_reviewed.pdf [file pone.0339012.s003.pdf]

PLOS ONE

Inoculation of indigenous nitrogen-fixers isolated from the Kuwait desert enhances seedling growth and nutrient uptake in a greenhouse bioassay

--Manuscript Draft--

**Manuscript Number:**	PONE-D-25-64029
**Article Type:**	Research Article
**Full Title:**	Inoculation of indigenous nitrogen-fixers isolated from the Kuwait desert enhances seedling growth and nutrient uptake in a greenhouse bioassay
**Short Title:**	Response to inoculation of indigenous diazotrophs in native seedlings
**Corresponding Author:**	Ali M Quoreshi, Ph.D<br>Kuwait Institute for Scientific Research<br>Kuwait, State of Kuwait KUWAIT
**Keywords:**	Free-living nitrogen-fixers; diazotrophs; inoculation; native plant restoration; nutrient uptake; biofertilizer
**Abstract:**	Desert soil degradation, primarily caused by anthropogenic disturbance and desertification, poses significant challenges for ecosystem restoration in degraded ecosystems. Soil microbial communities, particularly diazotrophs, play a crucial role in different soil system processes, including nutrient cycling, and can improve nitrogen-limited characteristics of nutrient-poor soil systems. Free-living and root-associated nitrogen-fixing bacteria have great potential to enhance nitrogen availability in the nitrogen-limited soil and support host plant growth and nutrient acquisition. Free-living nitrogen-fixing bacteria and root-associated rhizobacteria contribute a substantial amount of nitrogen to ecosystems, including arid lands. This study evaluated the growth performance and nutrient uptake ability of four native plant species of Kuwait inoculated with a consortium of selected indigenous putative diazotrophs isolated from the Kuwait desert. The seedlings of Vachellia pachyceras were inoculated with both indigenous root-nodule bacteria isolated from Kuwait desert and a commercial inoculum to evaluate their symbiotic efficiency. The seedlings were cultivated under greenhouse conditions in both native desert soils and in potting mix to assess the extent to which growth medium influenced inoculation response. The primary objective was to determine whether the inoculated indigenous N2 fixing bacteria could contribute early seedling development and nutrient acquisition, thereby supporting their potential use them as biofertilizer in future large-scale restoration efforts. Bacterial inoculation significantly enhanced plant dry mass and nutrient uptake across all tested plant species compared to the non-inoculated controls. The magnitude of improvement varied with bacterial density, plant species, and growth medium used.<br>These findings are consistent with evidence that isolated indigenous N2-fixers have the potential to enhance plant growth and nutrient uptake in selected native plant species, supporting their use as biofertilizers for restoration and revegetation efforts in arid environments. This study represents the first evaluation of Kuwait’s native seedlings inoculated with indigenous diazotrophs, highlighting their potential for sustainable ecosystem restoration.
**Order of Authors:**	Majda K. Suleiman
	Ali M Quoreshi, Ph.D
	Ali M. Quoreshi, Ph.D
	Anitha J. Manuvel
	Mini T. Sivadasan
	Sheena Jacob
**Additional Information:**	
**Question**	**Response**
**Financial Disclosure**	Yes

Powered by Editorial Manager® and ProduXion Manager® from Aries Systems Corporation

Enter a financial disclosure statement that describes the sources of funding for the work included in this submission. Review the submission guidelines for detailed requirements. View published research articles from *PLOS ONE* for specific examples.<br><br>This statement is required for submission and **will appear in the published article** if the submission is accepted. Please make sure it is accurate.<br><br>**Funded studies**<br>Enter a statement with the following details:<br>• Initials of the authors who received each award<br>• Grant numbers awarded to each author<br>• The full name of each funder<br>• URL of each funder website<br>• Did the sponsors or funders play any role in the study design, data collection and analysis, decision to publish, or preparation of the manuscript?<br><br>Did you receive funding for this work?	
Please add funding details.<br>as follow-up to "**Financial Disclosure**<br><br>Enter a financial disclosure statement that describes the sources of funding for the work included in this submission. Review the submission guidelines for detailed requirements. View published research articles from *PLOS ONE* for specific examples.<br><br>This statement is required for submission and **will appear in the published article** if the submission is accepted. Please make sure it is accurate.	This study was funded by Kuwait Foundation for the Advancement of Sciences (KFAS). The grand number is Project – P215-42SL-01

**Funded studies**<br>Enter a statement with the following details:<br>• Initials of the authors who received each award<br>• Grant numbers awarded to each author<br>• The full name of each funder<br>• URL of each funder website<br>• Did the sponsors or funders play any role in the study design, data collection and analysis, decision to publish, or preparation of the manuscript?<br><br>Did you receive funding for this work?"	
Please select the country of your main research funder (please select carefully as in some cases this is used in fee calculation).<br>as follow-up to "**Financial Disclosure**<br><br>Enter a financial disclosure statement that describes the sources of funding for the work included in this submission. Review the submission guidelines for detailed requirements. View published research articles from *PLOS ONE* for specific examples.<br><br>This statement is required for submission and **will appear in the published article** if the submission is accepted. Please make sure it is accurate.<br><br>**Funded studies**<br>Enter a statement with the following details:<br>• Initials of the authors who received each award<br>• Grant numbers awarded to each author<br>• The full name of each funder<br>• URL of each funder website<br>• Did the sponsors or funders play any role in the study design, data collection and analysis, decision to publish, or preparation of the manuscript?<br><br>Did you receive funding for this work?"	KUWAIT - KW
**Competing Interests**	The authors have declared that no competing interests exist.

Use the instructions below to enter a competing interest statement for this submission. On behalf of all authors, disclose any competing interests that could be perceived to bias this work—acknowledging all financial support and any other relevant financial or non-financial competing interests.<br><br>This statement is required for submission and **will appear in the published article** if the submission is accepted. Please make sure it is accurate and that any funding sources listed in your Funding Information later in the submission form are also declared in your Financial Disclosure statement.<br><br>View published research articles from *PLOS ONE* for specific examples.<br><br>**NO authors have competing interests**<br><br>Enter: *The authors have declared that no competing interests exist.*<br><br>**Authors with competing interests**<br><br>Enter competing interest details beginning with this statement:<br><br>*I have read the journal's policy and the authors of this manuscript have the following competing interests: [insert competing interests here]*<br><br>* typeset	
**Ethics Statement**<br><br>Enter an ethics statement for this submission. This statement is required if the study involved:<br><br>• Human participants<br>• Human specimens or tissue<br>• Vertebrate animals or cephalopods<br>• Vertebrate embryos or tissues	N/A

Powered by Editorial Manager® and ProduXion Manager® from Aries Systems Corporation

• Field research<br><br>Write "N/A" if the submission does not require an ethics statement.<br><br>General guidance is provided below. Consult the submission guidelines for detailed instructions. **Make sure that all information entered here is included in the Methods section of the manuscript.**	

Powered by Editorial Manager® and ProduXion Manager® from Aries Systems Corporation

**Format for specific study types**<br><br>**Human Subject Research (involving human participants and/or tissue)**<br>• Give the name of the institutional review board or ethics committee that approved the study<br>• Include the approval number and/or a statement indicating approval of this research<br>• Indicate the form of consent obtained (written/oral) or the reason that consent was not obtained (e.g. the data were analyzed anonymously)<br><br>**Animal Research (involving vertebrate animals, embryos or tissues)**<br>• Provide the name of the Institutional Animal Care and Use Committee (IACUC) or other relevant ethics board that reviewed the study protocol, and indicate whether they approved this research or granted a formal waiver of ethical approval<br>• Include an approval number if one was obtained<br>• If the study involved *non-human primates*, add *additional details* about animal welfare and steps taken to ameliorate suffering<br>• If anesthesia, euthanasia, or any kind of animal sacrifice is part of the study, include briefly which substances and/or methods were applied<br><br>**Field Research**<br><br>Include the following details if this study involves the collection of plant, animal, or other materials from a natural setting:<br>• Field permit number<br>• Name of the institution or relevant body that granted permission	
**Data Availability**<br><br>Authors are required to make all data underlying the findings described fully available, without restriction, and from the time of publication. PLOS allows rare exceptions to address legal and ethical concerns. See the PLOS Data Policy and FAQ for detailed information.	Yes - all data are fully available without restriction

Powered by Editorial Manager® and ProduXion Manager® from Aries Systems Corporation

A Data Availability Statement describing where the data can be found is required at submission. Your answers to this question constitute the Data Availability Statement and **will be published in the article**, if accepted.<br><br>**Important:** Stating 'data available on request from the author' is not sufficient. If your data are only available upon request, select 'No' for the first question and explain your exceptional situation in the text box.<br><br>Do the authors confirm that all data underlying the findings described in their manuscript are fully available without restriction?	
**Describe where the data may be found in full sentences. If you are copying our sample text, replace any instances of XXX with the appropriate details.**<br><br>• If the data are **held or will be held in a public repository**, include URLs, accession numbers or DOIs. If this information will only be available after acceptance, indicate this by ticking the box below. For example: *All XXX files are available from the XXX database (accession number(s) XXX, XXX.)*.<br>• If the data are all contained **within the manuscript and/or Supporting Information files**, enter the following: *All relevant data are within the manuscript and its Supporting Information files.*<br>• If neither of these applies but you are able to provide **details of access elsewhere**, with or without limitations, please do so. For example:<br><br>*Data cannot be shared publicly because of [XXX]. Data are available from the XXX Institutional Data Access / Ethics Committee (contact via XXX) for researchers who meet the criteria for access to confidential data.*<br><br>*The data underlying the results presented in the study are available from (include the name of the third party*	All relevant data are within the manuscript.

Powered by Editorial Manager® and ProduXion Manager® from Aries Systems Corporation

and contact information or URL).<br>• This text is appropriate if the data are owned by a third party and authors do not have permission to share the data.<br><br>* typeset	
Additional data availability information:	

Powered by Editorial Manager® and ProduXion Manager® from Aries Systems Corporation

Manuscript Click here to access/download;Manuscript;Manuscript.docx

Inoculation of indigenous nitrogen-fixers isolated from the Kuwait desert enhances seedling growth and nutrient uptake in a greenhouse bioassay

Majda K. Suleiman[1¶], Ali M. Quoreshi[1¶]*, Anitha J. Manuvel[1&], Mini T. Sivadasan[1&], Sheena Jacob[1&]

[1]Food Security Program, Environment and Life Sciences Research Center, Kuwait Institute for Scientific Research, Kuwait

Short title: Response to inoculation of indigenous diazotrophs in native seedlings

*Corresponding Author

E-mail: aquoreshi@kisr.edu.kw (AMQ)

[¶] These authors contributed equally to this work

[&] These authors also contributed equally to this work

Abstract:

Desert soil degradation, primarily caused by anthropogenic disturbance and desertification, poses significant challenges for ecosystem restoration in degraded ecosystems. Soil microbial communities, particularly diazotrophs, play a crucial role in different soil system processes, including nutrient cycling, and can improve nitrogen-limited characteristics of nutrient-poor soil systems. Free-living and root-associated nitrogen-fixing bacteria have great potential to enhance nitrogen availability in the nitrogen-limited soil and support host plant growth and nutrient acquisition. Free-living nitrogen-fixing bacteria and root-associated rhizobacteria contribute a substantial amount of nitrogen to ecosystems, including arid lands. This study evaluated the growth performance and nutrient uptake ability of four native plant species of Kuwait inoculated with a consortium of selected indigenous putative diazotrophs isolated from the Kuwait desert. The seedlings of *Vachellia pachyceras* were inoculated with both indigenous root-nodule bacteria isolated from Kuwait desert and a commercial inoculum to evaluate their symbiotic efficiency. The seedlings were cultivated under greenhouse conditions in both native desert soils and in potting mix to assess the extent to which growth medium influenced inoculation response. The primary objective was to determine whether the inoculated indigenous N_2 fixing bacteria could contribute early seedling development and nutrient acquisition, thereby supporting their potential use them as biofertilizer in future large-scale restoration efforts. Bacterial inoculation significantly enhanced plant dry mass and nutrient uptake across all tested plant species compared to the non-inoculated controls. The magnitude of improvement varied with bacterial density, plant species, and growth medium used.

These findings are consistent with evidence that isolated indigenous N_2-fixers have the potential to enhance plant growth and nutrient uptake in selected native plant species, supporting their use as biofertilizers for restoration and revegetation efforts in arid environments. This study represents the first evaluation of Kuwait's native seedlings inoculated with indigenous diazotrophs, highlighting their potential for sustainable ecosystem restoration.

Introduction

Recent land degradation in the Kuwait desert, resulting from prevailing environmental extremes and anthropogenic disturbances, is a significant environmental concern that contributes to further desertification and poor soil conditions. Consequently, degradation of native vegetation communities affects the characteristics of desert vegetation. Nevertheless, desert plants are indigenous plants adapted to the extreme local environment. They are characterized by slower growth and adaptation mechanisms that enable them to survive in harsh environments. However, it is extremely challenging to succeed in natural regeneration and revegetation in degraded soil conditions, as the soils have already lost their essential nutrient content, organic matter, and key biological soil properties, including the soil microbial community [1]. Evidences suggest that the native plants' population in Kuwait has been diminishing in recent years due to soil degradation, extreme weather, climatic fluctuations and various anthropogenic activities [2, 3]. Restoration and revegetation of the native plants are essential to protect them from extinction. To restore the native plant community successfully, it is important to recover soil fertility and the rhizosphere microbial community, as the soil is the major source of nutrients for plant growth and development.

Nevertheless, desert lands host diverse resilient microbial communities that exist as free-living, associate with plants, or form soil biocrusts [4].

The soil microbial communities are considered as the key driving force for regulating critical soil ecosystems processes, such as nutrient cycling and decomposition of organic matter, nutrient mineralization, and biogeochemical cycling of terrestrial ecosystems [5, 6, 7, 8]. The available soil nutrients for native plant are deficient in Kuwait's desert soil due to lack in organic matter (<1%), low clay materials, high in calcareous materials, and low in essential nutrients [9]. Availability of soil nutrients is one of the important factors for the restoration of the degraded desert soil. The extreme arid environmental conditions generally recognized that nutrient biogeochemical cycling, especially N cycling are microbially mediated [10]. Soil microbial community including N fixing bacteria could play an important role in nutrient cycling, decomposition of organic matter, and improvement of soil fertility, and maintenance of healthy ecosystems [11]. To plan effective strategies for conserving and restoring desert ecosystems, it is often necessary to manipulate and establish associated soil microorganisms for the successful establishment of vegetation cover.

Diazotrophic bacteria are a group of bacteria that dwell in the rhizospheric soil or are associated with the root system of plants and fix atmospheric dinitrogen to plant-available nutrients. These diazotrophs have great potential to alleviate nitrogen scarcity in nitrogen-scarce soils and enhance the accessibility of nitrogen to the host plant [12, 13, 14]. Bashan et al. [15] and Moreno et al. [16] demonstrated that the PGPB enhanced the growth of native plant species and improved soil stability in degraded forest and desert soils. Liu et al. [17] reported that these bacteria accelerated plant development and improved the survival rate of different forest seedlings. Inoculation of plant-growth-promoting (PGP) nitrogen-fixing

bacteria is an alternative to the use of chemical fertilizers [18]. The generous use of chemical fertilizers triggers environmental concern and economic losses. In view of the growing interest in developing alternative approaches to improve soil fertility, the use of bioinoculants and biofertilizers containing plant-beneficial microorganisms represents a promising alternative to chemical fertilizers. Bioinoculants have the potential to mobilize the essential nutrients to the plants [19]. Deficiency of available nitrogen to plants in the Kuwait's desert soil signifies the necessity to develop a strategy to enhance its availability via diazotrophic bacteria to improve the soil fertility [20]. Although numerous studies have documented the significant impact of plant growth-promoting bacteria (PGPB) and other beneficial microbes on crop yield and productivity, limited research has focused on their potential to enhance the growth and biomass of desert seedlings/plants through free-living or root-associated beneficial microorganisms [21].

Application of indigenous free-living diazotrophs and root-associated plant growth – promoting bacteria for promoting the growth of Kuwait native plants remains unexplored. In this current study, we evaluated the plant growth performance and nutrient uptake after inoculation of free-living nitrogen-fixing bacterial isolates and root-associated bacteria, obtained from our previous study [20] to *Rhanterium epapposum, Farsetia aegyptia, Haloxylon, Vachellia pachyceras Haloxylon salicornicum* under greenhouse condition. The hypothesis of this research was that inoculation of free-living and root-associated diazotrophs may increase growth and nutrient uptake of native desert plants under the current experimental setting. Therefore, our main objective of this study was to evaluate the potential of indigenous nitrogen-fixing bacteria isolates from Kuwait's desert soils to enhance seedling growth

performance and nutrient uptake of native desert plants under controlled greenhouse conditions.

Materials and methods

Inoculum preparation

The bacterial isolates used in this greenhouse experiment are indigenous diazotrophic strains isolated from the rhizospheric soil of *Rhanterium epapposum, Farsetia aegyptia,* and *Haloxylon salicornicum* (free-living nitrogen-fixing bacteria), as well as root nodules of *Vachellia pachyceras* (symbiotic nitrogen-fixers). The nitrogen-fixing capacity of isolated diazotrophs was previously tested and confirmed using the Acetylene Reduction Assay, and they were identified using the 16s rRNA gene sequencing method [20]. Totally, 6, 3, 11 and 19 strain of indigenous nitrogen-fixers were used as inoculum to test on the native plant species *Rhanterium epapposum, Farsetia aegyptia, Haloxylon salicornicum* and *Vachellia pachyceras*, respectively. For each bacterial isolate obtained from a single plant species, cell suspensions were prepared at a density of 10^8 CFU mL^{-1} [22, 23]. These isolates were then pooled together to form a mixture of bacterial inoculum with a final cell density of 10^8 CFU mL^{-1}. *From this mixture, a single dilution of* 10^4 *CFU* mL^{-1} *was prepared for Farsetia aegyptia, Rhanterium epapposum*, and *Haloxylon salicornicum* (free-living nitrogen-fixing bacteria). For Vachellia pachyceras, a dilution series of 10^8, 10^6, 10^4, and 10^2 CFU mL^{-1} was prepared.

Seedling Production

Two types of seedling growth media (desert soil and potting mix) were used in the experiment. Desert soil was collected from KSRI, packed in double autoclavable bags, and transported to the laboratory. Potting mix soil was prepared by mixing agricultural soil, peat moss, and

potting soil in a 2:1:1 volume-to-volume ratio (v/v) and packed into double autoclavable bags. The packed soils were sterilized twice in an autoclave at 121°C for 30 minutes, with complete cooling and mixing between cycles, and then air-dried. Likewise, jiffy pots and potting mix soil used in the initial seedling production were also sterilized prior to use. The irrigation water was sterilized once in an autoclave at 121°C for 15 min and used throughout the experiment. The sterilized jiffy pots were filled with autoclaved potting mix soil or desert soil and saturated with sterile water, and placed in a trey (30 jiffy pots). As Kuwait's native plant seeds were very sensitive to chemical treatment, the surface sterilization was done by soaking *Rhanterium epapposum* and *Farsetia aegyptia* overnight in sterile water and washed six times in sterile water, and one capitulum of *Rhanterium epapposum* was sown on the surface of the sterile potting mix soil or desert soil in jiffy pots. The seeds of *Farsetia aegyptia* were sown in jiffy pots filled with the potting mix or desert soil for germination. *Haloxylon salicornicum* seeds were washed six times with sterile water, and one seed was sowed on the top of the potting mix soil in jiffy pot for germination. *Vachellia pachyceras* seeds were treated with concentrated sulphuric acid for 30 min [24], washed six times in sterile water, and soaked in sterile water for overnight. The imbibed seeds were transferred to sterile petri-plate with moistened filter paper until germination. The germinated seeds were transferred to sterile jiffy pots with potting soil mix or desert sand. All the trays were maintained in the standard greenhouse conditions and irrigated with sterile water throughout the experiment as required.

Transplantation and Inoculation

Two-weeks after the germination in the jiffy pot, the seedlings of all the species were transplanted to one-gallon pots and maintained in the greenhouse. Six-weeks after the transplantation, the seedlings were inoculated with 20 ml of indigenous bacterial suspension

with desired cell densities (10^4 and 10^8 CFU mL^{-1} for *Rhanterium epapposum*, *Farsetia aegyptia*, *Haloxylon salicornicum* whereas 10^2, 10^4, 10^6 10^8 CFU mL^{-1} for *Vachellia pachyceras*) [25, 26]. Seedlings of all the species in the control treatment were not inoculated. Each treatment including control had eight seedlings as replicates which were arranged in Completely Randomized Design (CRD) in the greenhouse conditions. Furthermore, *Vachellia pachyceras* inoculated with 10 ml of 1 OD commercial American Type Culture Collection (ATCC) *Rhizobium leguminosarum* bv. viceae (10004; LOT: 59679216) and *Bradyrhizobium* sp. (BAA-1182; LOT: 60865886) in triplicate were also prepared and maintained along with the main experiment, to compare the potential of commercial inoculum to form nodulation and improve nitrogen uptake of *Vachellia pachyceras* under greenhouse conditions.

Data Collection and Data Analysis

The inoculated and non-inoculated control seedlings were maintained under greenhouse conditions, and their growth performance (plant height, number of leaves, stem diameter) and physical condition (vigor) were recorded before the experiment was terminated after the 10th month. At the end of the experiment, five randomly selected seedlings per treatment were excavated completely, and the soil was rinsed off the roots. The number and weight of the root nodules of *Vachellia pachyceras* were recorded. The seedlings were separated into roots and shoots and dried in an oven at 70°C for 48 hr or until a constant dry weight was achieved. The dry mass of roots and shoots of each plant species was determined on an analytical scale. The dried powdered shoot samples of each plant species were analyzed for Nitrogen (N), Phosphorus (P), and Potassium (K).

Seedling biomass, plant parameters and chemical analysis data were analyzed using Analysis of Variance Procedure (ANOVA) using SPSS® software - version 22 (IBM®) and means were compared using the Duncan's Multiple Range test to ascertain the significant differences among treatments at the 5% level of probability [27]. Type soil medium and bacterial inoculum were considered as factors in the analysis.

Results

Effect of potential nitrogen-fixer bacterial inoculations on plant growth parameters of selected native plant species

Rhanterium epapposum

Generally, inoculation with the two densities (10^4 and 10^8) of indigenous bacterial inoculum to *Rhanterium epapposum* significantly enhanced the average root ($p < 0.001$), shoot ($p < 0.001$), and total biomass ($p < 0.001$) per plant compared to the non-inoculated control (Table 1). Additionally, average root biomass per plant increased significantly ($p < 0.001$) in plants grown in potting soil when compared to that grown in desert soil (Table 1). However, bacterial inoculation and effect of growth medium types used had no significant impact on any other growth parameters determined when compared to the control (Table 1). The average root biomass of the seedlings grown in desert soil medium increased by 88% when compared to the seedlings grown in potting mix soil (Fig 1). Likewise, the indigenous bacterial inoculation increased average root biomass by 331% and 538%, average shoot biomass by 198% and 303% and average total biomass by 206% and 318% when inoculated with 10^4 CFU/ml and 10^8 CFU/ml densities (Fig 1). Although there was significant difference in the average root biomass and shoot biomass between non-inoculated control and those inoculated with indigenous bacteria, there was no significant difference within the two inoculum densities

used (10^4 CFU/ml and 10^8 CFU/ml) (Fig 1). Unlike the average root and shoot biomass, the average total biomass was significantly affected by inoculum density, which resulted in 54% increase in average total biomass of seedlings inoculated with indigenous inoculum of density 10^8 cells compared to those inoculated with cell density of 10^4 (Fig 1).

Fig 1. Effect of type of growth medium and density of bacterial inoculation on biomass per plant in *Rhanterium epapposum*.

Table 1. **Results of Analysis of Variance (P values) Testing for the Effect of Growth medium and Bacterial Inoculum on Growth Parameters of Selected Native Plant Species**

Variable	Plant Height	Stem Diameter	Plant Vigor	Root Biomass	Shoot Biomass	Total Biomass	Number of Root Nodules	Weight of Nodules	Root Shoot Ratio
Rhanterium epapposum									
Soil Media	0.499	0.386	0.584	0.011	0.496	0.769	NA	NA	0.150
Bacterial Inoculation	0.118	0.285	0.510	<0.001	<0.001	<0.001	NA	NA	0.894
Soil Media * Bacterial Inoculation	0.243	0.413	0.114	0.054	0.725	0.677	NA	NA	0.585
Farsetia aegyptia									
Soil Media	0.016	0.026	0.170	0.065	0.001	<0.001	NA	NA	0.546
Bacterial Inoculation	0.453	0.795	0.613	0.005	<0.001	<0.001	NA	NA	0.244
Soil Media * Bacterial Inoculation	0.194	0.413	0.613	0.987	0.335	0.332	NA	NA	0.872
Haloxylon salicornicum									
Soil Media	0.082	0.646	1.000	0.007	0.001	<0.001	NA	NA	0.009
Bacterial Inoculation	0.373	0.653	0.613	0.416	<0.001	<0.001	NA	NA	0.961
Soil Media * Bacterial Inoculation	0.138	0.414	0.243	0.428	0.046	0.050	NA	NA	0.907
Vachellia pachyceras									
Soil Media	0.653	0.366	0.057	0.893	0.481	0.787	0.806	0.934	0.200
Bacterial Inoculation	0.114	0.058	0.139	0.001	0.002	<0.001	0.009	0.010	0.131
Soil Media * Bacterial Inoculation	0.090	0.084	0.139	0.195	0.037	0.031	0.084	0.007	0.784

Farsetia aegyptia

Farsetia aegyptia, seedlings inoculated with the indigenous bacterial inoculum at 10^8 cells exhibited significantly greater root ($P \leq 0.005$), shoot ($P \leq 0.001$), and total biomass ($P \leq 0.001$) compared to those inoculated with 10^4 cells and the control (Table 1). Bacterial inoculum did not have any significant effect on other growth parameters.

Interestingly, plant height ($P \leq 0.016$), stem diameter ($P \leq 0.026$), shoot biomass ($P \leq 0.001$) and total biomass ($P<0.001$) of seedlings grown in desert soil was significantly higher than those grown in potting mix. Soil medium did not have significant effect on other growth parameters (Table 1).

Nevertheless, the average plant height, stem diameter, shoot biomass, and total biomass of *Farsetia aegyptia* seedlings grown in desert soil increased by 26%, 24%, 58%, and 58%, respectively, compared to seedlings grown in the potting-mix medium (Fig 2). A significant increase in shoot biomass (177%) and total biomass (182%) was observed in seedlings inoculated with the indigenous bacterial inoculum at a density of 10^8 CFU/ml cells compared to the control (Fig 2). Although inoculation with the 10^4 CFU/ml cell density also resulted in increases in these parameters, the improvements were not statistically significant relative to the control plants.

Fig 2. Effect of type of growth medium and density of bacterial inoculation on biomass per plant, plant height and stem diameter of *Farsetia aegyptia*.

Haloxylon salicornicum

Interestingly, the seedling growth substrate had a remarkable effect, significantly increased the average root biomass ($P \leq 0.007$), shoot biomass ($P \leq 0.001$), total biomass ($P < 0.001$), and root-to-shoot ratio ($P \leq 0.009$) of *Haloxylon salicornicum* seedlings (Table 1). Bacterial

inoculation at both 10^4 CFU/ml and 10^8 CFU/ml cell densities also significantly increased shoot biomass ($P \leq 0.001$), and total biomass ($P \leq 0.001$), compared to the non-inoculated control (Table 1). However, neither growth substrate nor bacterial inoculation significantly affected the other measured growth parameters compared to the control (Table 1).

The average root biomass, shoot biomass, and total biomass of *Haloxylon salicornicum* seedlings grown in desert soil increased by 476%, 46%, and 75%, respectively, compared with seedlings grown in the potting-mix medium (Fig 3). The average shoot biomass significantly increased by 89% and 123% in seedlings inoculated with indigenous bacterial inoculum at densities of 10^4 CFU/ml and 10^8 CFU/ml cells, respectively (Fig 3). Similarly, the average total biomass significantly increased by 95% and 123% in seedlings inoculated with 10^4 CFU/ml and 10^8 CFU/ml cell densities, respectively (Fig 3). However, there was no significant difference between the two inoculum densities with respect to shoot or total biomass.

Fig 3. Effect of type of growth medium and density of bacterial inoculation on biomass per plant of *Haloxylon salicornicum*.

Vachellia pachyceras

There was a significant increase in average root biomass ($P \leq 0.001$), shoot biomass ($P \leq 0.002$), total biomass ($P \leq 0.001$), number of root nodules ($P \leq 0.009$), and dry weight of root nodules ($P \leq 0.010$) in seedlings inoculated with the indigenous bacterial inoculum, with the 10^2 CFU/ml and 10^4 CFU/ml cell densities producing the most effective responses out of the four densities used i.e. 10^2, 10^4, 10^6 and 10^8 CFU/ml cells (Table 1 and Fig 4). However, neither inoculation nor growth media had a significant influence on the other recorded growth parameters (Fig 4).

The average root biomass increased by 190%, and 201% in seedlings inoculated with indigenous bacterial inoculum at densities of 10^2 CFU/ml and 10^4 CFU/ml cells, respectively (Fig 4). Similarly, the average shoot biomass increased by 124% and 106% the total biomass increased by 154% and 152% in seedlings inoculated with 10^2 CFU/ml and 10^4 CFU/ml cell densities, respectively (Fig 4). Interestingly, the highest concentration of indigenous bacterial inoculum (at 10^8 CFU/ml density) had no significant increase on average root biomass, shoot biomass and total biomass of the seedlings when compared to the non-inoculated control (Fig 4). The average number and dry weight of root nodules had significant effect in seedlings inoculated with indigenous bacterial inoculum at densities 10^2, 10^4 cells.

Fig 4. Effect of type of growth medium and density of bacterial inoculation on biomass per plant of *Vachellia pachyceras.*

Effect of putative nitrogen-fixer bacterial inoculation on plant nutrient uptake

In general, inoculation of putative nitrogen-fixing bacteria at various densities significantly affected the nutrient uptake of all four selected native plant species compared to the non-inoculated control (Table 2) under greenhouse experimental conditions. However, the increase in nutrient uptake ability of the tested bacterial inoculum varied depending on the density of bacteria in the inoculum used. In contrast, there was no significant interaction effect of growing media and inoculation on the nutrient uptake except in *Farsetia aegyptia* and *Haloxylon salicornicum* (Table 2).

Table 2. Results of Analysis of Variance (P values) Testing for the Effect seedling growth Medium and Bacterial Inoculation on N, P, K, Mg, Na, Ca, C and S uptake on Selected Native Plant Species

Variable	N	P	K	Mg	Na	Ca	C	S
Rhanterium epapposum								
Soil Type	0.573	0.102	0.342	0.531	0.160	0.056	0.717	0.988
Bacterial Inoculation	<0.001	0.001	<0.001	<0.001	0.001	0.001	<0.001	<0.001
Soil Type * Bacterial Inoculation	0.532	0.308	0.700	0.905	0.538	0.554	0.367	0.709
Farsetia aegyptia								
Soil Type	<0.001	0.189	0.010	0.463	0.655	0.011	0.013	0.004
Bacterial Inoculation	<0.001	<0.001	0.001	0.002	0.105	0.001	0.001	0.001
Soil Type * Bacterial Inoculation	0.181	0.197	0.126	0.221	0.062	0.372	0.540	0.167
Haloxylon salicornicum								
Soil Type	0.002	0.001	0.494	0.084	0.051	0.027	0.007	0.272
Bacterial Inoculation	0.014	0.023	0.002	0.001	0.007	0.001	0.004	0.011
Soil Type * Bacterial Inoculation	0.321	0.180	0.264	0.048	0.541	0.073	0.138	0.089
Vachellia pachyceras								
Soil Type	0.647	0.804	0.397	0.275	0.051	0.308	0.910	0.313
Bacterial Inoculation	0.022	0.042	0.032	0.015	0.039	0.029	0.026	0.040
Soil Type * Bacterial Inoculation	0.125	0.186	0.292	0.506	0.086	0.229	0.198	0.131

N: Nitrogen; P: Phosphorus; K: Potassium; Mg: Magnesium; Na: Sodium; Ca: Calcium; C: Carbon; S: Sulphur.

Rhanterium epapposum

Inoculation of *Rhanterium epapposum* with two densities (10^4 CFU/ml and 10^8 CFU/ml) of putative nitrogen-fixing bacterial inoculum in the rhizosphere significantly increased shoot

nitrogen (N) ($P \leq 0.001$), phosphorus (P) ($P \leq 0.001$), and potassium (K) ($P \leq 0.001$) content compared to the non-inoculated control (Table 2). Specifically, shoot N content increased by 176% and 319%, shoot P content by 185% and 244%, and shoot K content by 215% and 358% when inoculated with 10^4 CFU/ml and 10^8 CFU/ml cell densities, respectively, using the mixture of isolated indigenous putative nitrogen-fixers (Fig 5). The cell density of 10^8 CFU/ml produced the best results.

Fig 5. Effect of bacterial inoculation on N, P, K uptake measured as shoot N, P, K content per plant in *Rhanterium epapposum, Haloxylon salicornicum and Farsetia aegyptia.*

Additionally, inoculation also significantly enhanced shoot magnesium (Mg) ($P \leq 0.001$), sodium (Na) ($P \leq 0.001$), calcium (Ca) ($P \leq 0.001$), carbon (C) ($P \leq 0.001$), and sulfur (S) ($P \leq 0.001$) content (Table 2). However, neither the type of growth medium nor its interaction with bacterial inoculation had a significant effect on nutrient uptake (Table 2).

Farsetia aegyptia

Inoculation of *Farsetia aegyptia* with putative nitrogen-fixing bacterial inoculum in the rhizosphere significantly increased shoot nitrogen (N) ($P \leq 0.001$), phosphorus (P) ($P \leq 0.001$), and potassium (K) ($P \leq 0.001$) uptake compared to the non-inoculated control (Table 2). Inoculation with the 10^8 cell density resulted in significant increases in shoot N, P, and K by 180%, 207%, and 165%, respectively (Fig 5). Furthermore, inoculation also significantly enhanced shoot uptake of magnesium (Mg) ($P \leq 0.002$), calcium (Ca) ($P \leq 0.001$), carbon (C) ($P \leq 0.001$), and sulfur (S) ($P \leq 0.001$) (Table 2).

Interestingly, the seedling growth media also had a significant effect on shoot nutrient status, influencing N ($P \leq 0.001$), K ($P \leq 0.010$), Ca ($P \leq 0.011$), C ($P \leq 0.013$), and S ($P \leq$

0.004) content of the plant shoot (Table 2). However, the growth media had no significant effect on other parameters tested, including shoot P, Mg, and Na content (Table 2).

Haloxylon salicornicum

A similar trend was observed in *Haloxylon salicornicum* as the above mentioned native plant species. Shoot nitrogen (N) ($P \leq 0.014$), phosphorus (P) ($P \leq 0.023$), and potassium (K) ($P \leq 0.002$) content increased significantly in seedlings inoculated with the indigenous bacterial inoculum compared to the non-inoculated control (Table 2). As shown in Figure 5, inoculation increased shoot N content by 70% and 95%, shoot P content by 68% and 115%, and shoot K content by 84% and 148% when inoculated with 10^4 CFU/ml and 10^8 CFU/ml cell densities, respectively. There was no significant difference between the effects of 10^4 and 10^8 cell densities on the shoot N and K content. In addition to N, P, and K, bacterial inoculation also significantly enhanced shoot Mg ($P \leq 0.001$), Na ($P \leq 0.007$), Ca ($P \leq 0.001$), C ($P \leq 0.004$), and S ($P \leq 0.011$) content (Table 2).

The growth media had a significant positive effect on several nutrient parameters. Seedlings grown in desert soil showed higher shoot N ($P \leq 0.002$), Ca ($P \leq 0.027$), and C ($P \leq 0.007$) contents (Table 2), whereas those grown in potting soil mix recorded significantly higher shoot P ($P < 0.001$). In contrast, the growth media had no significant influence on shoot K, Mg, Na, or S contents (Table 2).

Vachellia pachyceras

Inoculation of *Vachellia pachyceras* seedlings with different densities of indigenous bacterial inoculum resulted in significant increases in shoot N ($P \leq 0.022$), P ($P \leq 0.042$), K ($P \leq 0.032$), Mg ($P \leq 0.015$), Na ($P \leq 0.039$), Ca ($P \leq 0.029$), C ($P \leq 0.026$), and S ($P \leq 0.040$) contents (Table 2). Among the inoculum densities, the 10^2 CFU/ml level produced the greatest

enhancement, increasing shoot N, P, and K contents by 152%, 129%, and 193%, respectively (Fig 6). Similar trends observed in shoot N, P, and K content and increased by 136%, 161%, and 141%, respectively when inoculated with 10^4 CFU/ml density of bacterial inoculum (Fig 6). No significant effects of growth media or their interaction with bacterial inoculation were observed for any of the measured shoot nutrients (Table 2).

Fig 6. Effect of bacterial inoculation on N, P, K uptake measured as shoot N, P, K content per plant in *Vachellia pachyceras.*

Evaluation of Commercial Inoculum on *Vachellia pachyceras*

The growth and nutrient uptake of *V. pachyceras* were also evaluated, after inoculation with the commercial ATCC bacterial strains, following the same procedure used for the indigenous bacterial inoculum experiments. The results are presented in Table 3.

Table 3. Results of Analysis of Variance (P values) Testing for the Effect of Commercial Inoculum on Nodulation, Growth Parameters, and Nitrogen Content of *Vachellia pachyceras*

Variable	Number of Nodules	Plant Height	Shoot Biomass	N Content
Soil Media	0.009	0.001	0.002	0.001
Inoculation	0.091	0.114	0.022	0.017
Soil Media * Inoculation	0.187	0.121	0.355	0.347

The seedlings grown in potting soil mix significantly increased average plant height by 75%, ($P \leq 0.001$), shoot biomass by 197%, ($P \leq 0.002$), number of nodules by 394% ($P \leq 0.009$), and nitrogen content by 247% ($P \leq 0.001$) compared to those grown in desert soil (Fig 7 and 8). The *Vachellia pachyceras* seedlings, when inoculated with commercial *Rhizobium*

leguminosarum ATCC and *Bradyrhizobium* sp. ATCC significantly increased the average shoot biomass ($P \leq 0.022$) and nitrogen ($P \leq 0.017$) content when compared to the non-inoculated control.

Fig 7. Effect of type of growth medium and commercial inoculum on shoot biomass and nitrogen content per plant of *Vachellia pachyceras.*

Fig 8. Effect of type of growth medium and commercial inoculum on number of nodules and plant height of *Vachellia pachyceras.*

The average shoot biomass of the *Vachellia pachyceras* seedlings significantly increased by 204% and 145% when inoculated with *Rhizobium leguminosarum* ATCC and *Bradyrhizobium* sp. ATCC, respectively, when compared to the non-inoculated control (Fig 7. Likewise, the average nitrogen content of *Vachellia pachyceras* seedlings significantly increased by 229% and 138% when inoculated with *Rhizobium leguminosarum* ATCC and *Bradyrhizobium* sp. ATCC, respectively, compared to the non-inoculated control (Fig 7).

Discussion

Achieving natural regeneration or even establishing planted seedlings in degraded desert lands is extremely challenging, particularly in areas with highly disturbed soils, unprotected environments, and poor physicochemical properties, alongside diminishing conditions of their functional microbial communities. The current research evaluated the influence of inoculating indigenous free-living diazotrophs and root-associated plant growth-promoting bacteria on the growth performance and nutrient uptake ability of four native plant species. The indigenous bioinoculants used in this study were isolated from the rhizosphere soils associated with three native keystone plant species, along with rhizobacteria obtained from *Vachellia pachyceras* root nodules in earlier experiment [20]. Overall our findings, demonstrate that

inoculation of indigenous bacterial strains across a range of inoculum densities significantly improved nutrient uptake in all four native plant species grown under controlled greenhouse conditions. These improvements were accompanied by corresponding increase in biomass production, indicating that the inoculation treatment contributed to plant growth. However, the magnitude, direction, and specificity of the responses depend among plant species, inoculum density, and growth medium, highlighting the importance of optimizing inoculum characteristics for different revegetation context Several studies have isolated and characterized beneficial microbes, including mycorrhizal fungi and plant-growth promoting rhizibacteria from arid and semi-arid soils; most of these works have remained descriptive or focused largely on agricultural crop species [22, 28, 29, 30]. However, information on the practical application of such beneficial microorganisms to enhance the growth and nutrient acquisition of native desert plants in the Middle Eastern regions remains limited [5, 20]. The current study contributes to filling this gap by demonstrating the potential of locally adapted microbial inoculants to improve the early establishment of native plant species, offering a promising strategy for restoring degraded desert landscapes.

The significant ($p \leq 0.001$) biomass responses observed at both inoculum densities (10^4 CFU/ml and 10^8 CFU/ml) indicate that *R. epapposum* responded positively to inoculation, likely through enhanced nitrogen acquisition and possibly improved root system functioning or effective nutrient mobilization. Interestingly, the difference between the two inoculum densities was not significant for either root or shoot biomass, although total plant biomass for the 10^8 CFU/ml treatment exceeded that of the 10^4 CFU/ml treatment by 54%. These results indicate that once a functionally effective threshold density is reached, additional increases in inoculum load do not confer further benefits in total biomass growth in *R. epapposum*. In the

case of *Farsetia aegyptia*, a similar pattern of enhanced growth response was observed, with markedly greater biomass accumulation, particularly at the higher inoculum density (10^8 CFU/ml), resulting in a 177% to 182% increase in shoot and total biomass compared to non-inoculated controls ($p \leq 0.001$). Similarly, *H. salicornicum* exhibited a substantial increase in seedling biomass at both inoculum densities, with total biomass increasing by 123% compared to non-inoculated controls ($p \leq 0.001$). Collectively, these results demonstrate that native desert plant species in Kuwait are highly responsive to inoculation with indigenous bacterial consortia. The observed increases in biomass across multiple species emphasize the potential of regionally adapted microbial inoculants to improve early seedling performance and promote growth under nutrient-limited desert conditions.

Vachellia pachyceras was examined across four inoculum densities (10^2, $10^{4,}$ $10^{6,}$ and 10^8 CFU/ml). Interestingly, in the case of *V. pachyceras* in the Fabaceae family, seedlings inoculated with low to medium densities (10^2 CFU/ml and 10^4 CFU/ml) exhibited significantly greater increase in root, shoot, and total biomass, as well as enhanced nodulation (nodule number and dry weight) compared to non-inoculated controls. In contrast, the highest inoculum density (10^8 CFU/ml) did not produce comparable improvements in growth or nodulation. The highest increases in root, shoot, and total biomass were observed by 190%, 124%, and 154%, respectively, under the lower inoculum concentrations.

The lower response at the highest density (10^8 CFU/ml) implies potential negative feedback, and this observation suggests a possible saturation effect at higher bacterial concentrations. Excessive cell concentration may limit nodulation efficiency and colonization due to oxygen constrains, resource competition, or interference among microbial populations. Biological nitrogen fixation is an energetically expensive process in which atmospheric N_2 is

converted into ammonia utilizing ATP, which requires carbon investment from the host plant [31]. The high inoculum densities may impose a carbon burden that outweighs the benefits of additional bacterial cells. This may explain reduced growth response in *Valhalla sp*. at 10^8 CFU/ml treatment level.

These findings are consistent with previous observations reporting density-dependent outcomes in legume-rhizobia symbioses [15, 28, 32, 33, 34], reinforcing the significance of plants optimizing inoculum density to achieve maximum plant benefit. In the context of restoration and revegetation programs, the results indicate that moderate inoculum densities are sufficient to enhance seedling establishment while maintaining cost-effectiveness.

In our experiment, the growth medium substantially affected root biomass across the evaluated species, with significant differences observed in *R. epapposum* ($p \leq 0.011$) and *H. salicornicum* ($p \leq 0.007$). The magnitude of biomass enhancement in certain species was particularly noteworthy. Increases in root systems are crucial in arid environments, where a well-developed root system enhances the plant's ability to access limited water and nutrient resources. In our study, the increase in average root biomass by 88% in *R. epapposum* when grown in desert soils compared to potting mix soils, while *H. salicornicum* showed a remarkable 476% increase under the same comparison. These results indicate a strong species-dependent response, and also highlights the influence of substrate quality, environment, nutrient availability, soil structure, and species native ecological growing conditions, which may play a key role in enabling root growth, proliferation, and nutrient uptake, and the performance of microbial inoculation. For *H. salicornicum* and *F. aegyptia*, desert soil supported superior growth, likely reflecting their evolutionary adaptation to native soil conditions and more compatible interactions with indigenous microbial communities.

However, effective plant growth enhancement also depends on successful root colonization by inoculated rhizobacteria, a process that is strongly shaped by the particular ecological niche and the degree of co-adaptation between host and microbial partners [35, 36].

Our results suggest that the success of inoculation may depend on a conducive soil environment that allows roots to colonize and support bacterial symbionts. Pankievicz et al. [31] indicated that effective symbiosis depends on the compatibility between a suitable host plant and diazotrophic bacteria, which is associated with favorable environmental conditions that support optimal nitrogen fixation. Inoculation with indigenous inoculum obtained from rhizospheric desert soil significantly improved plant growth, demonstrating that isolated native bacteria are better adapted to arid soil conditions and remain effective in low-nutrient environments, as observed in a greenhouse experiment.

Across all species, bacterial inoculation significantly increased plant nutrient content, particularly N, P, K, and key micronutrients (Mg, Ca, Na, and S). These increases likely reflect both direct microbial contributions to nutrient supply and indirect effects, including improved root development and i rhizosphere nutrient mobilization. Our study results are in agreement with previous findings showing that plant growth-promoting rhizobacteria enhance growth and nutrient uptake of maize plants when inoculated under greenhouse conditions [37, 38], and improved plant establishment of native plants and soil quality in degraded forest and desert soils [15, 16, 17]. Similarly, in a recent report demonstrated that isolated nitrogen-fixing bacteria from giant reed, and switch grass have the potential to influence plant growth and total nutrient uptake when inoculated to agricultural crops [39]. Similarly, PGPR-based inoculants and biostumulants have been reported to improve wheat growth [40], crop yield and nutrient uptake [41], and N, P, and K uptake of maize grown in nutrient-deficient calcisol

soil [28]. Tsegaye et al. [29] further demonstrated that single and consortium PGPR treatments significantly improved teff growth, grain yield and uptake of N, P, K, Ca, and S. Consistence with Egamberdiyeva [28] and Xu et al. [39], our findings confirm that inoculation enhances not only macronutrient uptake but also the accumulation of micronutrients Mg, Na, Ca, C, and trace elements.

In *R. epapposum*, inoculation significantly ($P \leq 0.001$) increased N, P, and K uptake by 176-319%, 185-244%, and 215-358%, respectively ($P \leq 0.001$), with the highest inoculum density yielding the greatest response. Neither growth medium nor its interaction with inoculation influenced nutrient uptake (Table 2), indicating that the bacterial effect was consistent across the soil typess. *F. aegyptia,* exhibited similar inoculation-induced increases in nutrient uptake as observed for increased biomass production. Moreover, in this species, growth medium significantly influenced N, K, Ca, and S uptake, indicating that the soil's inherent nutrient or physiochemical traits and compatibility with indigenous microbial communities influenced the plant's nutritional responses. The superior inoculation effect at 10^8 CFU/ml treatment implies that a sufficiently high bacterial load may be necessary to overcome competition or establishment barriers. These results suggest that optimizing inoculum density and matching soil conditions are critical for maximizing benefits in *F. aegyptia*. These outcomes suggest that the isolates possess full or partial symbiotic capabilities—either forming functional nodules and contributing to N fixation or promoting growth through indirect mechanisms, such as phytohormone production, nutrient solubilization, or root architecture modification. The diminished response at high bacterial density may reflect density-dependent regulation, competition, or suppression effects

In *H. salicornicum,* inoculation enhanced nutrient uptake significantly by 70-95% for N, 68-115% for P, and 84-148% for K, along with increased assimilation of Mg, ca, Na, C, and S. These broad improvements indicate that inoculation may facilitate mobilization of multiple macro- and micronutrients in nutrient-poor desert soils. The greater response at 10^8 CFU/ml versus 10^4 CFU/ml treatment suggests that higher inoculum density is beneficial in this species.

In contrast, *V. pachyceras,* showed maximum nutrient uptake at lower inoculum density (10^2 - 10^4 CFU/ml). While inoculation significantly elevated shoot N, P, K, Mg, Na, Ca, C, and S, the highest density (10^8 CFU/ml) did not significantly increase N. P, or K uptake. The growth medium and its interactions with inoculation did not have a significant effect on nutrient uptake. The responses in *Vachellia* system indicate a more complex symbiotic system in which isolated root-nodulating bacteria establish effective associations with the host plant primarily at low to moderate densities, likely achieving optimal root colonization and balanced plant-microbe interactions. Such responses suggest that these isolates possess full or partial symbiotic capabilities either forming functional nodules and contributing directly to nitrogen fixation or promoting plant growth via indirect mechanisms such as phytohormore production, nutrient solubilization, or root architecture modifications. The lack of positive response at higher bacterial density may reflect a possible density–dependent regulation, competition, or suppression. Both commercial and indigenous rhizobial inoculants significantly enhanced the growth and nutrient uptake of *V. pachyceras* seedlings under greenhouse conditions. Inoculation with commercial strains of *Rhizobium leguminosarum* ATCC and *Bradyrhizobium* sp. ATCC increased shoot biomass and nitrogen content compared to non-inoculated controls, with *R. leguminosarum* ATCC showing the greatest

response (Table 3), indicating the formation of effective symbioses and high N-fixation potential. The growth medium further influenced plant performance, as seedlings grown in potting soil exhibited greater height, shoot biomass, nodule number, and nitrogen content than those grown in desert soil, likely reflecting enhanced nutrient availability and more favorable physicochemical conditions. While greenhouse results demonstrate the promise of these commercial inoculants, field validation is necessary to determine their effectiveness under natural arid-land conditions. Overall, this greenhouse inoculation study confirms that inoculation with nitrogen-fixing bacteria can substantially enhance the growth and nutrient acquisition of desert native plants in nutrient-poor soils, as well as in nursery substrates. These findings are consistent with previous studies showing microbial inoculants increase not only N but also P, K, and micronutrient accumulation, likely through mechanisms such as improved root architecture, increased root exudation, nutrient solubilization, and rhizosphere activation, and phytohormone production [42, 43, 44, 45]. Although the specific mechanisms operating in this study cannot be conclusively identified within the scope of this study, previous studies attribute similar improvements to plant growth promotion, biological N fixation, organic compounds production, disease suppression, and root elongation [28, 46]. Other studies have reported that PGPB can suppress soil-borne pathogens and improve soil structure and microbial diversity, thereby indirectly supporting nutrient absorption and seedling establishment [18, 19]. Increased total nutrient uptake may also reflect increased shoot and root biomass as observed in agricultural crops [47, 48]. To our knowledge, this is the first study to evaluate growth response and nutrient mobilization of *V. pachyceras* seedlings inoculated with potential nitrogen-fixing rhizobacteria from the Kuwait desert, highlighting their potential application as biofertilizers for native plants establishment.

Conclusion

In general, both indigenous and commercial rhizobial inoculants seedling growth and nutrient uptake compared to non-inoculated seedlings under the current experimental conditions. The study evaluated the collective effects of isolated N-fixing bacteria rather than individual strains, demonstrating that growth improvements, successful nodulation, and N-fixation depend on host-microbe compatibility, inoculum density, and species-specific responses. The inoculation protocol proved effective across the tested native species, highlighting the potential of these isolates to improve growth and nutrient acquisition in Kuwait's desert flora. While the controlled nursery conditions offer important insights into symbiotic performance, they cannot fully capture the complexity of arid field environments, including heterogeneous soils, resident microbial competition, and environmental stresses. Therefore, field trials are required to validate the performance, ecological adaptability, and nitrogen-fixation efficiency of indigenous and commercial inoculants. Such research will support the development of optimized, field-ready bioinoculation strategies for sustainable restoration and revegetation efforts in desert ecosystems.

Acknowledgement

The authors immensely acknowledge the Kuwait Institute for Scientific Research (KISR) and Kuwait Foundation for the Advancement of Sciences (KFAS) for the constant support and encouragement throughout the project. We also thank the greenhouse helpers for their assistance in the maintenance of greenhouse experiments and laboratory helpers for laboratory analysis.

Author Contributions

Conceptualization: M. K. Suleiman, A. M. Quoreshi.

Formal analysis- laboratory and greenhouse: A. J. Manuvel, M. T. Sivadasan.

Statistical analysis: Sheena Jacob

Funding acquisition: M. K. Suleiman, A. M. Quoreshi

Investigation: M. K. Suleiman, A. M. Quoreshi, A. J. Manuvel, M. T. Sivadasan, Sheena Jacob

Methodology: M. K. Suleiman, A. M. Quoreshi, A. J. Manuvel, M. T. Sivadasan.

Project administration: M. K. Suleiman.

Supervision: M. K. Suleiman, A. M. Quoreshi.

Validation: M. K. Suleiman, A. M. Quoreshi.

Writing – original draft: M. K. Suleiman, A. M. Quoreshi, A. J. Manuvel.

Writing – review & editing: M. K. Suleiman, A. M. Quoreshi, A. J. Manuvel, M. T. Sivadasan, Sheena Jacob.

References

1. Lund PA. Multiple chaperonins in bacteria–why so many? FEMS microbiology reviews. 2009 Jul 1;33(4):785-800.
2. Omar SA, Bhat NR. Alteration of the Rhanterium epapposum plant community in Kuwait and restoration measures. International Journal of Environmental Studies. 2008 Feb 1;65(1):139-55.
3. Al-Shehabi Y, Murphy K. Flora richness as an indicator of desert habitat quality in Kuwait. Journal of Threatened Taxa. 2017 Feb 26;9(2):9777-85.

4. Coleine C, Delgado-Baquerizo M, DiRuggiero J, Guirado E, Harfouche AL, Perez-Fernandez C, et al. Dryland microbiomes reveal community adaptations to desertification and climate change. The ISME journal. 2024 Jan 1;18(1):wrae056.
5. Quoreshi AM, Suleiman MK, Kumar V, Manuvel AJ, Sivadasan MT, Islam MA, et al. Untangling the bacterial community composition and structure in selected Kuwait desert soils. Applied Soil Ecology. 2019 Jun 1;138:1-9.
6. Zhou H, Zhang D, Jiang Z, Sun P, Xiao H, Yuxin W, et. al. Changes in the soil microbial communities of alpine steppe at Qinghai-Tibetan Plateau under different degradation levels. Science of the Total Environment. 2019 Feb 15;651:2281-91.
7. Madouh TA, Quoreshi AM. The function of arbuscular mycorrhizal fungi associated with drought stress resistance in native plants of arid desert ecosystems: A review. Diversity. 2023 Mar 8;15(3):391.
8. Zhang C, Lei S, Wu H, Liao L, Wang X, Zhang L, Liu G, Wang G, Fang L, Song Z. Simplified microbial network reduced microbial structure stability and soil functionality in alpine grassland along a natural aridity gradient. Soil Biology and Biochemistry. 2024 191:109366.
9. Abdal MS, Suleiman MK. Soil conservation as a concept to improve Kuwait environment. Archives of Nature Conservation & Landscape Research. 2002 Jan 1;41(3-4):125-30**.**
10. Ramond JB, Jordaan K, Díez B, Heinzelmann SM, Cowan DA. Microbial biogeochemical cycling of nitrogen in arid ecosystems. Microbiology and Molecular Biology Reviews. 2022 Jun 15;86(2):e00109-21.

11. Anthony MA, Bender SF, Van Der Heijden MG. Enumerating soil biodiversity. Proceedings of the National Academy of Sciences. 2023 Aug 15;120(33):e2304663120.

12. Mehnaz S, Kowalik T, Reynolds B, Lazarovits G. Growth promoting effects of corn (Zea mays) bacterial isolates under greenhouse and field conditions. Soil Biology and Biochemistry. 2010 Oct 1;42(10):1848-56.

13. Núñez JD, Muñoz D, Planelles R, Grau JM, Artero F, Anriquez A, et al. Effects of inoculation with Azospirillum brasilense on the quality of Prosopis juliflora seedlings. Forest Systems. 2012 Nov 28;21(3):364-72.

14. Ricci F, Rossetto Marcelino V, Blackall LL, Kühl M, Medina M, Verbruggen H. Beneath the surface: community assembly and functions of the coral skeleton microbiome. Microbiome. 2019 Dec 12;7(1):159.

15. Bashan Y, Salazar BG, Moreno M, Lopez BR, Linderman RG. Restoration of eroded soil in the Sonoran Desert with native leguminous trees using plant growth-promoting microorganisms and limited amounts of compost and water. Journal of environmental management. 2012 Jul 15;102:26-36.

16. Moreno M, De-Bashan LE, Hernandez JP, Lopez BR, Bashan Y. Success of long-term restoration of degraded arid land using native trees planted 11 years earlier. Plant and soil. 2017 Dec;421(1):83-92.

17. Liu L, Zhang T, Gilliam FS, Gundersen P, Zhang W, Chen H, et al. Interactive effects of nitrogen and phosphorus on soil microbial communities in a tropical forest. PloS one. 2013 Apr 12;8(4):e61188.

18. Saeed Q, Xiukang W, Haider FU, Kučerik J, Mumtaz MZ, Holatko J, Naseem M, Kintl A, Ejaz M, Naveed M, Brtnicky M. Rhizosphere bacteria in plant growth promotion, biocontrol, and bioremediation of contaminated sites: a comprehensive review of effects and mechanisms. International journal of molecular sciences. 2021 Sep 29;22(19):10529.
19. Sun W, Shahrajabian MH, Soleymani A. The roles of plant-growth-promoting rhizobacteria (PGPR)-based biostimulants for agricultural production systems. Plants. 2024 Feb 23;13(5):613.
20. Suleiman MK, Quoreshi AM, Bhat NR, Manuvel AJ, Sivadasan MT. Divulging diazotrophic bacterial community structure in Kuwait desert ecosystems and their N2-fixation potential. PLoS one. 2019 Dec 26;14(12):e0220679.
21. Saleem M, Nawaz F, Hussain MB, Ikram RM. Comparative effects of individual and consortia plant growth promoting bacteria on physiological and enzymatic mechanisms to confer drought tolerance in maize (Zea mays L.). Journal of Soil Science and Plant Nutrition. 2021 Dec;21(4):3461-76.
22. Kifle MH, Laing MD. Isolation and screening of bacteria for their diazotrophic potential and their influence on growth promotion of maize seedlings in greenhouses. Frontiers in Plant Science. 2016 Jan 8;6:1225.
23. Idris A, Labuschagne N, Korsten L. Efficacy of rhizobacteria for growth promotion in sorghum under greenhouse conditions and selected modes of action studies. The Journal of Agricultural Science. 2009 Feb;147(1):17-30.

24. Suleiman MK, Dixon K, Commander L, Nevill P, Bhat NR, Islam MA, Jacob S, Thomas R. Seed germinability and longevity influences regeneration of Acacia gerrardii. Plant ecology. 2018 May;219(5):591-609.

25. Diouf D, Forestier S, Neyra M, Lesueur D. Optimisation of inoculation of Leucaena leucocephala and Acacia mangium with rhizobium under greenhouse conditions. Annals of forest science. 2003 May 1;60(4):379-84.

26. Al-Shaharani TS, Shetta ND. Evaluation of growth, nodulation and nitrogen fixation of two acacia species under salt stress. World Applied Sciences Journal 13 (2): 256-265, 2011.

27. Little TM, Hills FJ. Agricultural experimentation. Design and analysis. 1978 Jan 1.

28. Egamberdiyeva D. The effect of plant growth promoting bacteria on growth and nutrient uptake of maize in two different soils. Applied soil ecology. 2007 Jun 1;36(2-3):184-9.

29. Tsegaye Z, Alemu T, Desta FA, Assefa F. Plant growth-promoting rhizobacterial inoculation to improve growth, yield, and grain nutrient uptake of teff varieties. Frontiers in Microbiology. 2022 Oct 21-13:896770.

30. Alonazi MA, Alwathnani HA, Al-Barakah FN, Alotaibi F. Native Plant Growth-Promoting Rhizobacteria Containing ACC Deaminase Promote Plant Growth and Alleviate Salinity and Heat Stress in Maize (Zea mays L.) Plants in Saudi Arabia. Plants. 2025 Apr 2;14(7):1107.

31. Pankievicz VC, Irving TB, Maia LG, Ané JM. Are we there yet? The long walk towards the development of efficient symbiotic associations between nitrogen-fixing bacteria and non-leguminous crops. BMC biology. 2019 Dec 3;17(1):99.

32. Postma J, Hok-A-Hin CH, Voshaar JO. Influence of the inoculum density on the growth and survival of Rhizobium leguminosarum biovar trifolii introduced into sterile and non-sterile loamy sand and silt loam. FEMS Microbiology Ecology. 1990 Jan 1;6(1):49-57.
33. Burghardt LT, Epstein B, Hoge M, Trujillo DI, Tiffin P. Host-associated rhizobial fitness: dependence on nitrogen, density, community complexity, and legume genotype. Applied and environmental microbiology. 2022 Aug 9;88(15):e00526-22.
34. Rahman A, Manci M, Nadon C, Perez IA, Farsamin WF, Lampe MT, et al. Competitive interference among rhizobia reduces benefits to hosts. Current Biology. 2023 Jul 24;33(14):2988-3001.
35. Hassan MK, McInroy JA, Kloepper JW. The interactions of rhizodeposits with plant growth-promoting rhizobacteria in the rhizosphere: a review. Agriculture. 2019 Jul 4;9(7):142.
36. Rilling JI, Acuña JJ, Nannipieri P, Cassan F, Maruyama F, Jorquera MA. Current opinion and perspectives on the methods for tracking and monitoring plant growth–promoting bacteria. Soil Biology and Biochemistry. 2019 Mar 1;130:205-19.
37. ElJiati A, Elmaati Y, Ouchaou H. Plant Growth Promoting Rhizobacteria (PGPR) Isolated from an Arid Soil in Saudi Arabia Improve Maize Growth. Atlas Journal of Biology. 2024 May 4:830-8.
38. Kuan KB, Othman R, Abdul Rahim K, Shamsuddin ZH. Plant growth-promoting rhizobacteria inoculation to enhance vegetative growth, nitrogen fixation and nitrogen remobilisation of maize under greenhouse conditions. PloS one. 2016 Mar 24;11(3): e0152478.

39. Xu J, Kloepper JW, Huang P, McInroy JA, Hu CH. Isolation and characterization of N2-fixing bacteria from giant reed and switchgrass for plant growth promotion and nutrient uptake. Journal of basic microbiology. 2018 May;58(5):459-71.
40. Buisset E, Soust M, Scott PT. The Isolation of Free-Living Nitrogen-Fixing Bacteria and the Assessment of Their Potential to Enhance Plant Growth in Combination with a Commercial Biostimulant. Microbiology Research. 2025 Mar 18;16(3):69.
41. Kumar A, Maurya BR, Raghuwanshi R, Meena VS, Tofazzal Islam M. Co-inoculation with Enterobacter and Rhizobacteria on yield and nutrient uptake by wheat (Triticum aestivum L.) in the alluvial soil under indo-gangetic plain of India. Journal of plant growth Regulation. 2017 Sep;36(3):608-17.
42. Vacheron J, Desbrosses G, Bouffaud ML, Touraine B, Moënne-Loccoz Y, Muller D, et al. Plant growth-promoting rhizobacteria and root system functioning. Frontiers in plant science. 2013 Sep 17;4:356.
43. Elhaissoufi W, Ghoulam C, Barakat A, Zeroual Y, Bargaz A. Phosphate bacterial solubilization: A key rhizosphere driving force enabling higher P use efficiency and crop productivity. Journal of Advanced Research. 2022 May 1;38:13-28.
44. Chen W, Wang J, Chen X, Meng Z, Xu R, Duoji D, Zhang J, He J, Wang Z, Chen J, Liu K. Soil microbial network complexity predicts ecosystem function along elevation gradients on the Tibetan Plateau. Soil Biology and Biochemistry. 2022 Sep 1;172:108766.
45. Francioli D, Strack T, Dries L, Voss-Fels KP, Geilfus CM. Roots of resilience: Optimizing microbe-rootstock interactions to enhance vineyard productivity. Plants, People, Planet. 2025 May;7(3):524-35.

46. Chanway CP. Plant growth promotion by Bacillus and relatives. Applications and systematics of Bacillus and relatives. 2002 Aug 9:219-35.

47. Adesemoye AO, Torbert HA, Kloepper JW. Increased plant uptake of nitrogen from 15N-depleted fertilizer using plant growth-promoting rhizobacteria. Applied Soil Ecology. 2010 Sep 1;46(1):54-8.

48. Saubidet MI, Fatta N, Barneix AJ. The effect of inoculation with Azospirillum brasilense on growth and nitrogen utilization by wheat plants. Plant and soil. 2002 Aug;245(2):215-22.

Figure Click here to access/download;Figure;Fig 1.tiff

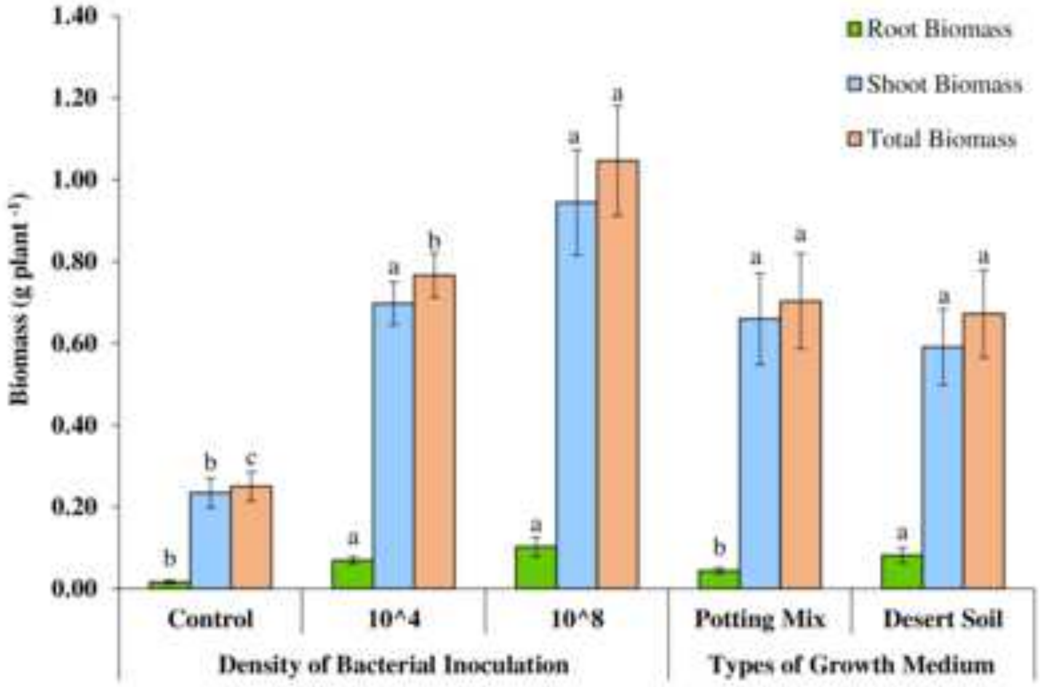

Fig 1. Effect of type of growth medium and density of bacterial inoculation on biomass per plant in *Rhanterium epapposum*.

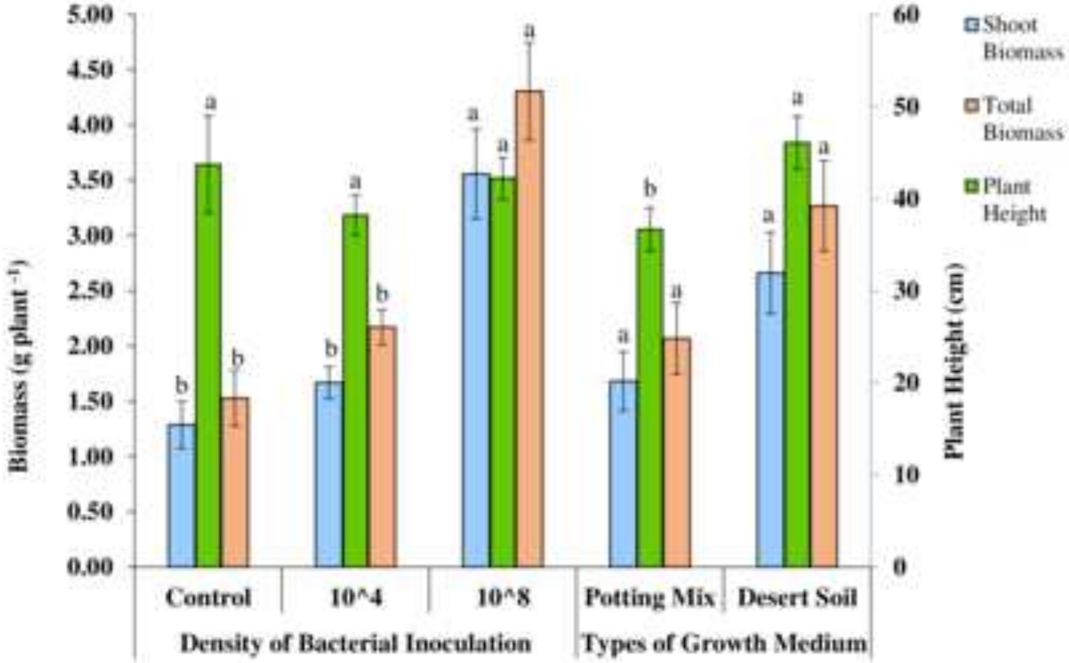

Fig 2. Effect of type of growth medium and density of bacterial inoculation on biomass per plant, plant height and stem diameter of *Farsetia aegyptia*.

Figure
Click here to access/download;Figure;Fig 3.tiff

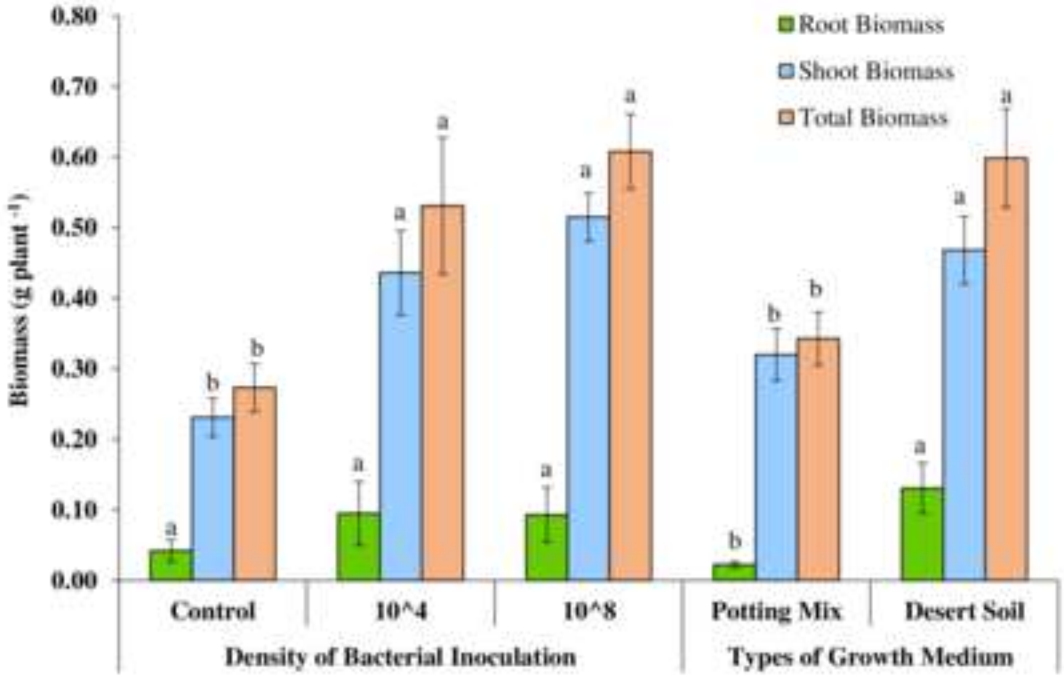

Fig 3. Effect of type of growth medium and density of bacterial inoculation on biomass per plant of *Haloxylon salicornicum*.

Figure Click here to access/download;Figure;Fig 4.tiff

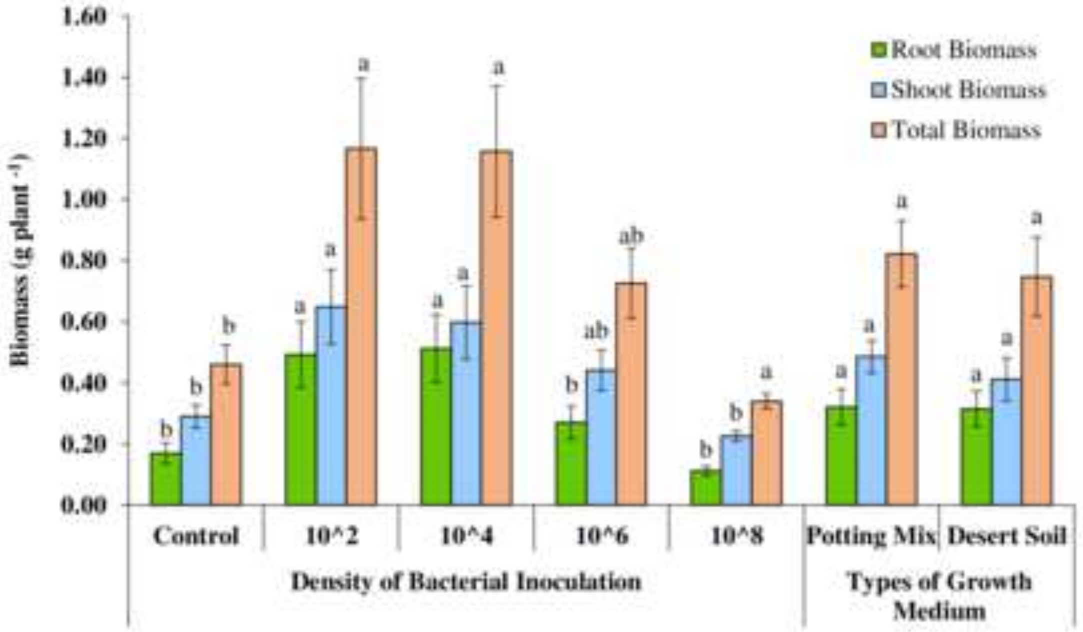

Fig 4. Effect of type of growth medium and density of bacterial inoculation on biomass per plant of *Vachellia pachyceras*.

 Click here to access/download;Figure;Fig 5.tiff

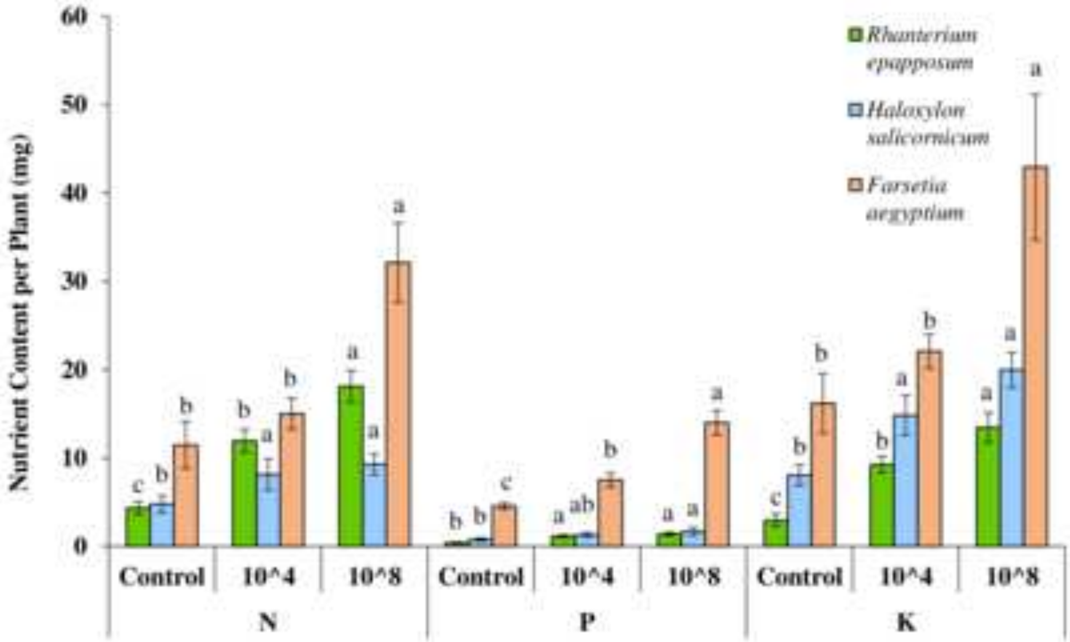

Fig 5. Effect of bacterial inoculation on N, P, K uptake measured as shoot N, P, K content per plant in *Rhanterium epapposum, Haloxylon salicornicum and Farsetia aegyptia.*

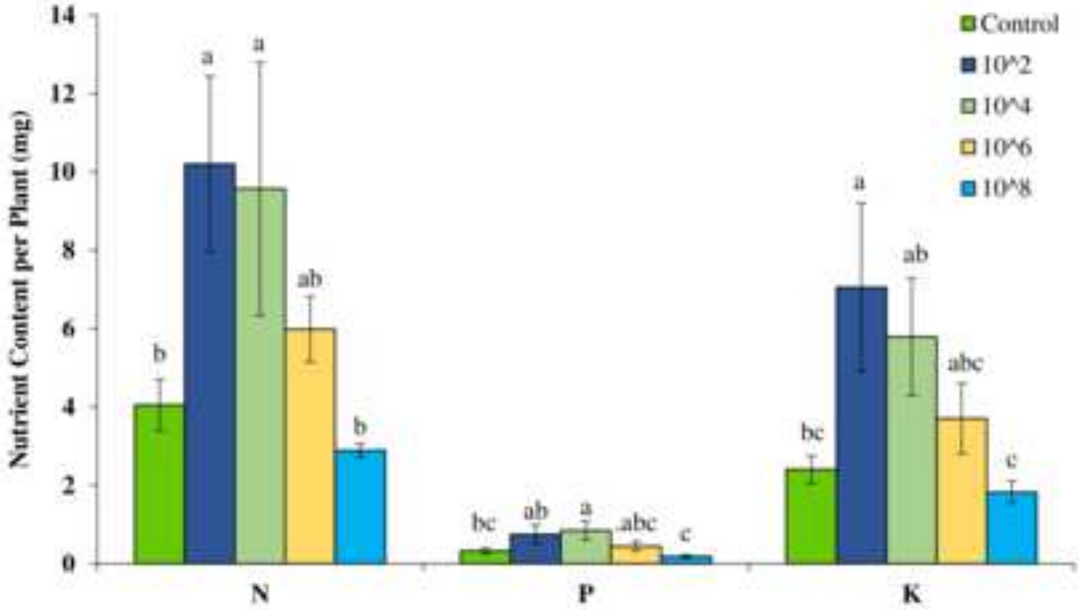

Fig 6. Effect of bacterial inoculation on N, P, K uptake measured as shoot N, P, K content per plant in *Vachellia pachyceras*.

Figure Click here to access/download;Figure;Fig 7.tiff

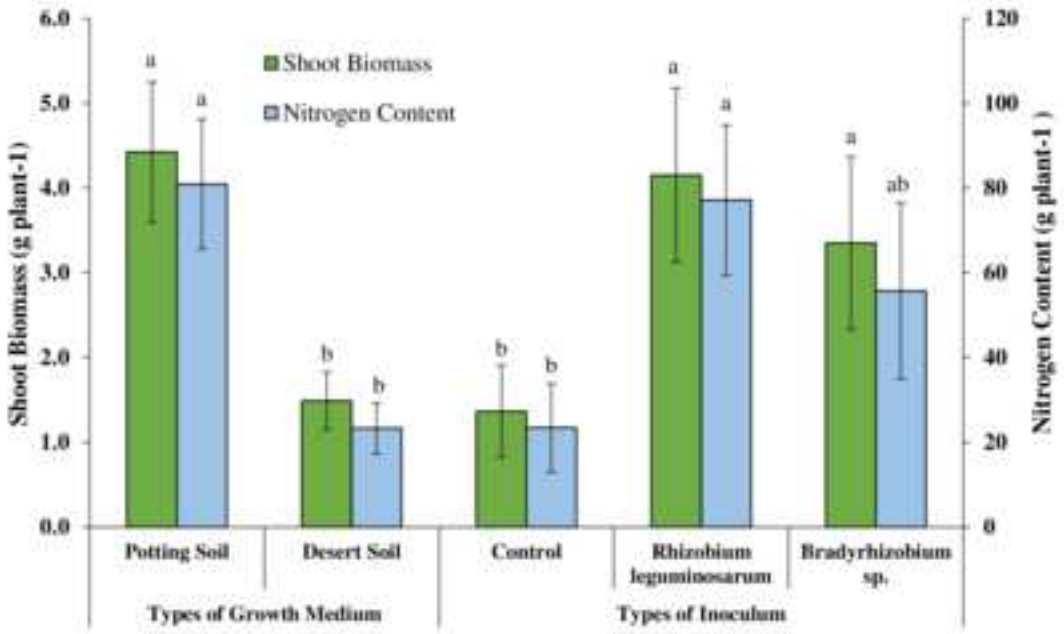

Fig 7. Effect of type of growth medium and commercial inoculum on shoot biomass and nitrogen content per plant of *Vachellia pachyceras*.

Figure
Click here to access/download;Figure;Fig 8.tiff

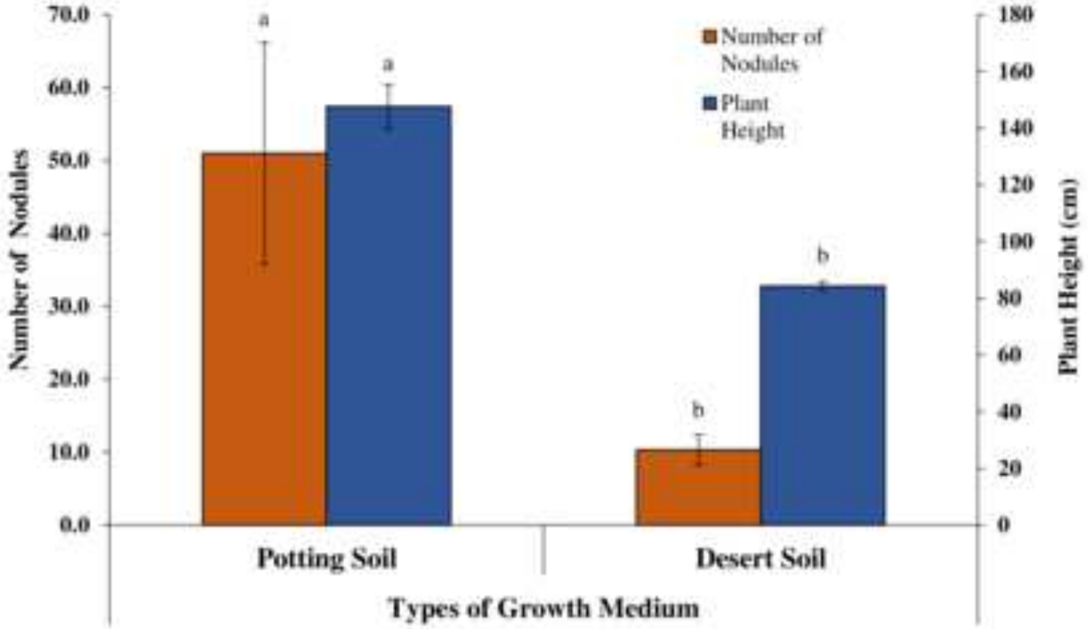

Fig 8. Effect of type of growth medium and commercial inoculum on number of nodules and plant height of *Vachellia pachyceras*.
