# Supplementary material for: Inoculation with indigenous nitrogen-fixers enhances seedling growth and nutrient uptake in a greenhouse bioassay
Source: PLoS One. 2026 Apr 15;21(4):e0339012. doi: 10.1371/journal.pone.0339012 (PMC13082604; doi:10.1371/journal.pone.0339012)
Supplement: Attachment — Submitted filename: Response to Reviewers (Final revision)_ALI_March 5.docx [file pone.0339012.s005.docx]

***Inoculation of indigenous nitrogen-fixers isolated from the Kuwait desert enhances seedling growth and nutrient uptake in a greenhouse bioassay***

***Revised Title:*** “*Inoculation with indigenous nitrogen-fixers enhances seedling growth and nutrient uptake in a greenhouse bioassay*”

**First Revision comments**

**General Comments (from e-mail)**

When submitting your revision, we need you to address these additional requirements.

**Comment 1.** Please ensure that your manuscript meets PLOS ONE's style requirements, including those for file naming. The PLOS ONE style templates can be found at

[https://journals.plos.org/plosone/s/file?id=wjVg/PLOSOne_formatting_sample_main_body.pdf](https://linkprotect.cudasvc.com/url?a=https%3a%2f%2fjournals.plos.org%2fplosone%2fs%2ffile%3fid%3dwjVg%2fPLOSOne_formatting_sample_main_body.pdf&c=E,1,7Z_u1wVgIMo692GNOXXKp4HKvHVRIqxUSSqjcKPgCeqL9EBK_4q8KenFSLCKZs6TVzfjvN96AA4jZ4zmhixWv1LJ68AXr_XBHecQpntTpeZ8Npo,&typo=1&ancr_add=1) and

[https://journals.plos.org/plosone/s/file?id=ba62/PLOSOne_formatting_sample_title_authors_affiliations.pdf](https://linkprotect.cudasvc.com/url?a=https%3a%2f%2fjournals.plos.org%2fplosone%2fs%2ffile%3fid%3dba62%2fPLOSOne_formatting_sample_title_authors_affiliations.pdf&c=E,1,1jC68uBEng97kEg1BhbvVsdbAyMnaKGqR_6KYZUWkZtRte7oDwBaEDIDIaiqosoF3LBZWzLzVLPN-Ir1gS3Bu1bUdi25wpgF3Zt-k_wcT9CF11McnU0,&typo=1&ancr_add=1)

***Response 1:*** Thank you for your suggestion. The revised manuscript has been carefully reviewed and now complies with all PLOS ONE’s style requirements outlined in the links provided above.

**Comment 2.** In your Methods section, please provide additional information regarding the permits you obtained for the work. Please ensure you have included the full name of the authority that approved the field site access and, if no permits were required, a brief statement explaining why.

***Response 2:*** The desert soil used as growth medium in this greenhouse assay was collected from the KISR’s Station for Research and Innovation (KSRI). No permit was required for site access sample collection, as the research station is owned and operated by Kuwait Institute for Scientific Research (KISR).

**Comment 3.** Thank you for stating the following financial disclosure:

“This study was funded by Kuwait Foundation for the Advancement of Sciences (KFAS). The grand number is Project – P215-42SL-01”

Please state what role the funders took in the study.  If the funders had no role, please state: "The funders had no role in study design, data collection and analysis, decision to publish, or preparation of the manuscript."

If this statement is not correct you must amend it as needed.

Please include this amended Role of Funder statement in your cover letter; we will change the online submission form on your behalf.

***Response 3:*** As mentioned, the following statement was added in the revised cover letter and uploaded.

“The funders had no role in study design, data collection and analysis, decision to publish, or preparation of the manuscript.”

**Comment 4.** If the reviewer comments include a recommendation to cite specific previously published works, please review and evaluate these publications to determine whether they are relevant and should be cited. There is no requirement to cite these works unless the editor has indicated otherwise.

***Response 4:*** Thanks for the comment. We carefully reviewed concern citation. The current citations in the revised manuscript are correct and relevant.

**Comment 5.** Review Comments to the Author

Please use the space provided to explain your answers to the questions above. You may also include additional comments for the author, including concerns about dual publication, research ethics, or publication ethics. (Please upload your review as an attachment if it exceeds 20,000 characters)

Reviewer #1: The manuscript and data presented is quite interesting but the writing or presentation is wanting. I have left my review comments in the attached manuscript file. Most glaring issues are as follows: The standard rules of writing scientific names are not followed in the document. The introduction section is not clear but contains information that is jumbled up with no clear flow from the general background, to the problem at hand, to the research gap and the aim of the study. The results presented in the tables should indicate the means plus or minus the standard deviations assuming the measurements were conducted in triplicates as should be the case. The labels in the figures are not legible. Figures 7 and 8 are a bit chaotic because they contain different variables with different measurement units but are graphed together e.g plant height and number of nodules in Figure 8. This shouldn't be the case at all. The rest of the comments are in the attachment.

***Response 5 (a):*** We sincerely thank the reviewer for the constructive and insightful comments. All remarks and the reviewer comments in the annotated manuscript have been carefully addressed in the revised version. Specifically, scientific names have been thoroughly reviewed and corrected throughout the manuscript to ensure compliance with standard nomenclature conventions. The introduction section has been substantially reorganized, revised thoroughly, removed repetitions to improve clarity and logical flow, clearly establish background to research problems, identified knowledge gaps, and objective of the study.

Regarding the results section, the data provided in the tables are p values derived from statistical analyses. Therefore, the values presented in these tables should not present as mean ± standard deviation as well as presenting units are not relevant. Additional statistical analysis was performed as commented and all the figures have been improved and presented in a clearer and more appropriate format in the revised manuscript.

We believe these revisions have significantly strengthened the manuscript.

Reviewer #2: This manuscript provides valuable and novel insights into the use of indigenous diazotrophic bacteria to enhance the growth and nutrient uptake of native plant species in arid ecosystems. The topic aligns well with restoration ecology priorities and is technically sound.

Major Strengths:
- Novel and relevant contribution to arid-land restoration
- Strong experimental design with factorial approach
- Clear evidence of treatment effects supported by data
- Practical implications for restoration and biofertilizer applications

***Response (5b):***

We sincerely thank the reviewer for the positive evaluation of our manuscript and for recognizing its novelty, methodological strength, and practical relevance. We greatly appreciate the acknowledgment of its contribution to arid-land restoration and biofertilizer applications. Your encouraging feedback is highly valued.

**Major Points for Improvement:**
1. Mechanistic interpretations are inferred. Authors should clarify limitations and suggest future validation.

***Response:*** The result section was carefully revised to avoid any mechanistic interpretations and presented in the revised manuscript

2. Statistical assumptions (normality/variance) are not reported. It is recommended to include.

***Response*:** Prior to analysis, data normality and homogeneity was verified. As recommended this information is now reported in the Data Collection and Data Analysis section.

3. Desert soil physicochemical properties are not provided. It is suggested that a soil properties table be added.

***Response:*** Thank you for this valuable suggestion. The desert soil used in this study was collected from the KSRI. As recommended, a table summarizing the soil physiochemical properties of the soil has now been added to the Supplementary Materials Section (Table S2) in the revised manuscript.

4. Composition of inoculum mixtures not fully detailed. It is suggested to include isolate IDs and genera.
***Response:*** Thanks for the helpful comment. The isolate IDs corresponding genera used in this experiment were previously published in our earlier study and are cited in the inoculum preparation section of the manuscript. To improve clarity and completeness, we have now added a supplementary table detailing the composition of the inoculum mixture including isolate ID and respective genera in the Supplementary Materials Section (Table S1).

**Minor Comments:**
- Standardize formatting of CFU units, superscripts, and species italics

***Response:*** We apologize for the oversight. All CFU units, superscripts, and scientific names have been carefully reviewed and corrected to ensure consistence and adherence to standard formatting conventions throughout the revised manuscript.

- Improve clarity of figure axis labels

***Response:*** The figure axis labels have been revised and improved to enhance the clarity and readability in the revised manuscript.

- Minor grammatical smoothing recommended

***Response:*** As recommended, the manuscript has been thoroughly proofread and minor grammatical issues have been corrected to improve overall clarity and flow.

**More specific section wise comments are as follows: (From e-mail)**

*Line number mentioned below are reflected as per original submission of the manuscript.*

**TITLE / ABSTRACT**

**Comment 6.** Page 1, line 1–2:
The title uses “enhances seedling growth and nutrient uptake in a greenhouse bioassay. “Consider adding 'indigenous nitrogen-fixers' earlier for indexing clarity, for example: 'Inoculation with indigenous nitrogen-fixers enhances seedling growth…”

***Response 6:*** We appreciate the suggestion on modifying the title for indexing clarity. As suggested the title has been revised to improve indexing clarity and now reads: “*Inoculation with indigenous nitrogen-fixers enhances seedling growth and nutrient uptake in a greenhouse bioassay*” in the revised manuscript.

**Comment 7.** Page 2, lines 24–28:
Abstract introduces desert soil degradation but repeats phrases such as “nutrient-poor soil systems”. Tighten to improve flow by merging lines 24–29 into a more concise problem definition.

***Response 7:*** Thank you for this helpful comment. We have revised the overall abstract, particularly line 24-29 were re-written to improve clarity and flow by eliminating repetitive phrasing and merging the indicated lines into a more concise problem statement in the revised manuscript.

**Comment 8.** Page 2, lines 39–40:
Typo in “thereby supporting their potential use them as biofertilizer…” Remove “them” “…supporting their potential use as biofertilizers…”

***Response 8:*** Corrected as suggested. The sentence now read “…. supporting their potential use as biofertilizer.”

**INTRODUCTION**

**Comment 9.** Page 3, lines 52–63:
Very descriptive and slightly repetitive regarding desertification and native vegetation decline. Condense by removing repeated causes (climate, anthropogenic effects) already mentioned above.

***Response 9:*** Thank you for your suggestions. Introduction section has been thoroughly revised, condensed to remove repetition of previously states drivers of desertification throughout the introduction section, while keeping the key contextual information.

**Comment 10.** Page 3, line 61–62:
“soil microbial community [1]”. Citation [1] does not appear to support soil microbial loss specifically. Ensure citations align with the stated claim or modify wording.

***Response 10:*** We agree and revised the sentence to better reflect the cited reference, where appropriated.

**Comment 11.** Page 4, lines 82–85:
Diazotroph definition could be streamlined; sentences are long. This can be revised, for example: “Diazotrophs are rhizospheric or root-associated bacteria capable of fixing atmospheric nitrogen into plant-available forms.”

***Response 11:*** As suggested, the definition of diazotroph has been rewritten for clarity and conciseness and incorporated in the revised manuscript.

**Comment 12.** Page 5, lines 102–103:
“obtained from our previous study [20]” is useful, but no brief summary of identification outcomes. Add one sentence explaining how many strains and which genera were isolated to improve continuity.

***Response 12:*** We understand the reviewer’s concern regarding the identification of bacterial strains used in this study as well as to improve the continuity with the current study. To address this comment a table summarizing the details of bacterial isolates and their major genera used in this experiment has been added in the supplementary section (Table S1).

**MATERIALS & METHODS**

**Comment 13.** Page 6, lines 117–122:
Comment: Cell concentrations and pooling are described, but the genera/species identities of isolates are not listed. Add a supplemental table summarizing: strain code, genus, isolation source, and 16S accession numbers (if applicable).

***Response 13:*** As suggested, a supplementary table (Table S1) summarizing: strain code, genus, isolation source, and 16S accession numbers has been added in the revised manuscript.

**Comment 14.** Page 7, lines 132–138:
Soil preparation is described, but no soil physicochemical properties are provided. Add a soil property table (pH, EC, OM%, total N, P, texture), as these directly affect the interpretation of nutrient uptake.

***Response 14:*** As suggested, a new soil properties table has included in the revised manuscript as a supplementary table (Table S2) reporting the physiochemical properties of soil used for the experiment to support interpretation of nutrient uptake results.

**Comment 15.** Page 7, lines 144–151:
Seed sterilization and scarification methods are detailed but lack justification for differences across species. Add at least a sentence explaining why sulfuric acid treatment was used only for Vachellia pachyceras.

***Response 15:*** As suggested, a justification sentence has been added in the revised manuscript explaining why sulfuric acid scarification was applied only to *Vachellia pachyceras* due to its hard, impermeable seed coat and dormancy requirement.

**Comment 16.** Page 8, lines 160–167:
The commercial inoculum strains are listed only as ATCC numbers. Also provide scientific names, e.g., Rhizobium leguminosarum bv. viciae…

***Response16:*** Scientific names corresponding to the ATCC strains have added as per the brochure supplied along with the commercial bacterium, the commercial bacterial strains are cited as *Rhizobium leguminosarum* (ATCC^®^10004^TM^) and *Bradyrhizobium* sp. (ATCC^®^BAA-1182) in the revised manuscript.

**Comment 17.** Page 8, lines 171–182:
Comment: Statistical methods are adequate, but missing assumption verification details. Add information on the following tests:
• Shapiro–Wilk or KS test for normality
• Levene’s test for homogeneity of variances
• Effect size metrics (optional but recommended)

***Response 17:***
Thanks for your suggestion. A sentence addressing your concern has been added as” Prior to analysis, data normality was verified using Shapiro-Wilk test and homogeneity of variances using Levene’s Test” under Data Collection and Data Analysis Section in the revised manuscript.

**RESULTS**

**Comment 18.** Page 10–15, Tables 1 & 2:
Clear presentation, but units for biomass (e.g., g plant⁻¹) are not specified. Add units beneath column headers for clarity.

***Response 18:*** Regarding the comment on the Tables 1 and 2, the data provided in the tables are p values derived from statistical analyses only and not the actual biomass data. Therefore, the values presented in these tables (p values) should not present as mean ± standard deviation and also presenting units are not relevant.

**Comment 19.** Page 12, line 214–217:
“did not have any significant effect…” appears multiple times across species. Consider reporting non-significant trends if biologically relevant.

***Response 19:*** The result section has been revised to report non-significant trends wherever necessary and relevant.

**Comment 20.** Figures (p.11–18):
Figures lack clear axis labels (units for biomass, nutrient concentration). Add units (e.g., mg plant⁻¹ for nutrients, g for biomass).

***Response 20:*** As suggested, we carefully reviewed all figures for its axis accuracy, axis labels. The figures axis labels are correct reflecting appropriate measurement units. We corrected axis label in Figure 7 as suggested.

**DISCUSSION**

**Comment 21.** Page 19–21:
Very detailed, but occasionally repetitive on “biomass increased significantly…” Combine repetitive biomass comparisons and focus more on ecological interpretation.

***Response 21:*** As suggested, the discussion section has been thoroughly reviewed and revised to reduce repetitive reporting of biomass increases and strengthen ecological interpretation of the findings.

**Comment 22.** Page 21, lines 397–399:
Growth responses are linked to nitrogen acquisition, but no mechanisms were measured. Add a sentence acknowledging mechanistic limitations (e.g., no ARA post-inoculation, nifH expression).

***Response 22:*** We appreciate your comments and agreed mechanistic limitations. A limitation statement has been added in the revised manuscript noting that nitrogen fixation activity and nifH expression were not directly measured and should be evaluated in future work.

**Comment 23.** Page 22, lines 418–423:
“Biological nitrogen fixation is an energetically expensive process…”. It is a good context. However, add a citation to support the discussion of metabolic cost.

***Response 23:*** Thanks for your concern. We reviewed the sentence and stated as “Biological nitrogen fixation is an energetically expensive process in which atmospheric N_2_ is converted into ammonia utilizing ATP, which requires carbon investment from the host plant”. An appropriate reference was already sited (Pankievicz *et al*., 2019).

**Comment 24.** Page 24–26:
Discussion of nutrient uptake improvements is strong, but does not distinguish direct vs. indirect mechanisms. Add a 1–2 sentence hypothesis section:
• Direct: N fixation, P solubilization
• Indirect: phytohormones, root architecture changes

***Response 24:*** We appreciate reviewer concern. We thoroughly reviewed the paragraphs and has been revised by including hypothesized mechanisms distinguishing a complex symbiotic system that may operate as direct effects contributing directly to biological nitrogen fixation or promoting plant growth via indirect mechanisms such as phytohormore production, nutrient solubilization, or root architecture modifications.

**CONCLUSION**

**Comment 25.** Page 27, lines 535–546:
Strong applied relevance but lacks explicit limitations to improve realism and strengthen the case for future field trials. The following limitations can be added:
• Greenhouse vs. field performance
• Microbe persistence in natural soils
• Competition with native microbiota

***Response 25:*** We sincerely thank the reviewer for this valuable suggestion. In response, we have carefully reviewed and revised the Conclusion section to explicitly acknowledge key limitations in order to improve practical realism of the study and strengthen the rationale for further field validations. Specifically, we have added limitations related to Greenhouse vs. field performance, the persistence and stability of the introduced microbes in natural soils, and their competition with native microbiota. Future field validation is now explicitly recommended

**REFERENCE SECTION**

**Comment 26.** Page 28 onward:
Ensure consistency in formatting (journal names sometimes abbreviated, sometimes not). Adapt to PLOS ONE reference formatting guidelines.

***Response 26:*** The reference section has been carefully reviewed to ensure to PLOS ONE reference formatting guidelines. The reference list section thoroughly checked for consistency.

**Comments from PDF manuscript**

**Comment 27.** Lines 52 – 54: Recent land degradation in the Kuwait desert, resulting from prevailing environmental extremes and anthropogenic disturbances, is a significant environmental concern that contributes to further desertification and poor soil conditions

where are the recent citations to support this claim?

***Response 27:*** We appreciate your concern. Introduction section has been reviewed and revised. Appropriate references are cited accordingly. The references cited Omar and Bhat 2008, Al Shehabi and Murphy 2017 support the sentence mentioned claim.

**Comment 28.** Lines 67-68: Nevertheless, desert lands host diverse resilient microbial communities that exist as free living, associate with plants, or form soil biocrusts.

this should be the start of the next paragraph

***Response 28:*** We apologize for the mistake. In the revised manuscript this sentence has been shifted to the start of the next paragraph.

**Comment 29.** Line 69-81 (full paragraph): there is a mix up of information in this paragraph. talking about microbial communities then back to soil nutrients and their deficiency in Kuwait soils, then back to the soil microbes and N fixing bacteria. there is no flow of the presented information.

***Response 29:*** Thanks for your valuable comment. The mentioned full paragraph has been reviewed and revised as suggested to improve readability and logical flow and incorporated in the revised manuscript.

**Comment 30.** Line 82-101 (full paragraph): this should be mentioned together with the information on N fixing bacteria you have already mentioned in the previous paragraph. Otherwise, it looks like you are now mentioning N fixing bacteria here for the firs time. consolidate the information

***Response 30:*** The discussion of nitrogen-fixing bacteria has been revised and consolidated with the preceding paragraph to improve continuity and readability.

**Comment 31.** Line 86: PGPB

what is this? do not use abbreviations without defining them at first mention

***Response 31:*** We apologize for the mistake. The term has been defined at first mention in the revised manuscript as “Plant growth-promoting bacteria (PGPB).

**Comment 32.** Line 98: plant growth-promoting bacteria (PGPB)

this is what you should do at first mention

***Response 32:*** Noted and corrected as suggested.

**Comment 33.** Line 117-119: Rhanterium epapposum, Farsetia aegyptia, and Haloxylon salicornicum (free-living nitrogen-fixing bacteria), as well as root nodules of Vachellia pachyceras (symbiotic nitrogen-fixers).

observe the common rules of writing scientific names. after writing in full at first mention, all subsequent mentions should be done with the genus name abbreviated/initialized by the first letter

***Response 33:*** All scientific names mentioned in the manuscript has been thoroughly checked and names are now written in full at first mention and abbreviated thereafter accordingly to standard conventions in the revised manuscript.

**Comment 34.** Lines 127-129: *From this mixture, a single dilution of 10⁴ CFU mL⁻¹ was prepared for Farsetia aegyptia, Rhanterium epapposum*, and *Haloxylon salicornicum* (free-living nitrogen-fixing bacteria).

italicize only the **scientific names**

***Response 34*:** We apologize for the mistake. Formatting has been corrected. The scientific name are italicized in the revised manuscript.

**Comment 35.** Lines 129-130: For Vachellia pachyceras, a dilution series of 10⁸, 10⁶, 10⁴, and 10² CFU mL⁻¹ was prepared.

italicize all scientific names

***Response 35****:* As mentioned earlier, all scientific names were checked and revised throughout the manuscript for italicization and incorporated in the revised manuscript.

**Comment 36.** Line 133: KSRI

if this is an abbreviation then write in full at first mention. if not mentioned elsewhere in the document then just write in full and do away with the abbreviation

***Response 36****:* Yes, this is an abbreviation. KSRI is expanded as “KISR’s Station for Research and Innovation” at first mentioned and the abbreviation retained thereafter in the revised manuscript.

**Comment 37.** Line 136: 30 minutes

is this the standard period of autoclaving?

***Response 37****:* Standard period of autoclaving is 15 minutes for broth, liquid media etc. However, the time period for soil sample is extended to 30 minutes at 121 ^0^ C and autoclaving twice for 30 min each time to ensure proper sterilization of soil samples. This standard protocol has already been mentioned in the manuscript.

**Comment 38.** Line 163: OD

define all abbreviations at first use

***Response 38****:* As mentioned earlier, all abbreviations were defined at first use in the revised manuscript. As Optical density (OD).

**Comment 39.** Line 170-171: The inoculated and non-inoculated control seedlings were maintained under greenhouse conditions, and their growth performance……

specify the conditions e.g humidity, temperature, daylight period etc

***Response 39****:* The greenhouse conditions are 25 ± 2ºC, 60-70% relative humidity, and a 14-h photoperiod. These greenhouse conditions have been specified in the revised manuscript.

**Comment 40.** Table 1. Results of Analysis of Variance (P values) Testing for the Effect of Growth medium and Bacterial Inoculum on Growth Parameters of Selected Native Plant Species

can the values/means be presented as means plus or minus the standard deviations supposing the measurements were conducted in replicates which should be the case

***Response 40****:* As mentioned earlier under response 18, the comment on the Tables 1 and 2, the data provided in the tables are p values derived from statistical analyses only and not the actual biomass data. Therefore, the values presented in these tables (p values) should not present as mean ± standard deviation and also presenting units are not relevant.

**Comment 41.** Table 1: Plant Height

if it is possible you could include photos to illustrate the differences in plant height for all the tested plants against the different variable/treatments used.

***Response 41****:* Thanks for your suggestion. However, while we appreciate the suggestion, standardized photographic comparisons were not available for all treatments and for all the tested plants; therefore, quantitative data are retained.

**Comment 42.** Table 2. same comment as in the previous table

***Response 42****:* Refer to Response 40.

**Comment 43.** Line 316: Figure 5

all others are written as Fig. except this. maintain consistency

***Response 43****:* Figure 5 is corrected to Fig. 5 in the revised manuscript to maintain consistency.

**Comment 44.** Table 3. same comment as in the previous tables

***Response 44****:* Refer to Response 40.

**Comment 45.** Line 494: *V. pachyceras,*

this is how you should be writing all the scientific names all over the document after writing all of them in full at the first time

***Response 45****:* Noted and corrected in the revised manuscript.
